# A quantum-inspired, biomimetic, and fractal framework for self-healing AI code generation: bridging responsible automation and emergent intelligence

**DOI:** 10.3389/frai.2025.1662220

**Published:** 2025-11-07

**Authors:** Mohammadreza Nehzati

**Affiliations:** VMC MAR COM Inc. DBA HeyDonto, Knoxville, TN, United States

**Keywords:** self-healing AI systems, quantum-inspired optimization, biomimetic computing, fractal scalability, adaptive code generation

## Abstract

AI-powered code generation systems available today are ill-suited for deployment in agile software development contexts due to various limitations. The paper proposes a self-healing counterpart framework based on quantum-inspired optimization, biomimetic, and fractal principles to solve these fundamental issues. Our Quantum Solution Space Manager keeps more than one candidate solution in superposition states. In doing so, it achieves 94.7% code correctness (versus 87.3%) with respect to a leading approach. The biomimetic error detection system, inspired by biological immune mechanisms, has a sensitivity of 95.2 per cent, with a false-positive rate of 2.3 per cent. Effectively, 94.7 per cent of detected errors are automatically corrected. Fractal optimization allows for a considerable 89.4% success rate during cross-architectural propagation, while distributed intelligence networks allow different intelligences and agents to learn together. The framework is validated as effective through an analysis of 15,000 software engineering tasks across five domains. This helps reduce the critical error rate by 54% and the remaining development time by 41%, along with notable improvements in maintainability and security metrics. The results lay down the path for adaptive software development systems to create responsible automation and emergent intelligence.

## 1 Introduction

The rapid evolution of artificial intelligence has fundamentally transformed the landscape of software engineering, ushering in an era where automated code generation transcends traditional paradigms of human-machine collaboration (Alenezi and Akour, 2025; Sauvola et al., 2024). Contemporary software development increasingly relies on AI-powered tools that promise enhanced productivity, reduced development cycles, and improved code quality. However, despite remarkable advances in generative AI technologies, current code generation systems exhibit significant limitations in adaptability, error recovery, and scalable optimization—challenges that become particularly pronounced in dynamic, large-scale software environments where requirements evolve rapidly and system complexity grows exponentially.

The proliferation of AI-driven development tools, exemplified by GitHub Copilot, ChatGPT, and specialized code generation platforms, has demonstrated substantial potential in augmenting developer capabilities (Bird et al., 2023; France, 2024). Nevertheless, these systems predominantly operate through static pattern recognition and template-based generation, lacking the sophisticated self-correction mechanisms and adaptive intelligence required for robust, autonomous software development (Cámara et al., 2023). This limitation becomes critically apparent when considering the increasing demand for resilient software systems capable of self-modification, error detection, and automatic recovery—capabilities that mirror biological systems’ inherent adaptability and quantum systems’ superposition-based optimization principles (Ernst and Bavota, 2022).

The contemporary landscape of AI-assisted software development has witnessed unprecedented growth, with recent research demonstrating both the transformative potential and inherent limitations of current approaches. Tufano et al. (2024) introduced AutoDev, representing a significant advancement in automated AI-driven development through end-to-end workflow integration. Their work highlighted the importance of moving beyond isolated code snippet generation toward comprehensive development process automation. Similarly, Ridnik et al. (2024) proposed AlphaCodium, emphasizing the transition from traditional prompt engineering to sophisticated flow engineering methodologies, thereby addressing the need for more structured and predictable code generation processes. The empirical evaluation of existing tools reveals mixed results regarding their practical effectiveness. El Haji et al. (2024) conducted comprehensive studies on GitHub Copilot’s test generation capabilities in Python, uncovering significant limitations in generating comprehensive test suites and handling edge cases. These findings align with broader observations by Zhang et al. (2023), who identified substantial gaps between user expectations and actual tool performance, particularly in complex software engineering scenarios requiring deep contextual understanding and long-term code maintenance considerations. Odeh et al. (2024) provided a comparative analysis of various AI techniques for automated code generation, revealing that current approaches primarily rely on large language models trained on vast code repositories. While these models demonstrate impressive pattern recognition capabilities, they lack the adaptive mechanisms necessary for dynamic optimization and self-correction. The research emphasized the need for more sophisticated frameworks that can learn from execution feedback and adapt to changing requirements without extensive retraining.

Owing to these limitations, processes based on AI code generation may not have practical effects, as observed in a review of current literature. To identify the issues that affect the existing works, we can highlight the following. First, the existing works mainly rely on static pattern matching and do not include any dynamic adaptation mechanisms. This is evident from the relatively deterministic (i.e., static) nature of transformer-based models (Odeh et al., 2024). The restrictions on classic data science become painfully apparent in the challenging enterprise software engineering problem, where the quality of the solution depends on context-dependent optimization rather than statistical correlation. Moreover, the simultaneous requirement of a lot of human time to debug and assure quality in these systems conflicts with the logic of AI-driven technology production (El Haji et al., 2024). Additionally, existing multi-agent approaches focus on task allocation rather than the emergence of collective intelligence, leading to missed opportunities for collaborative learning and knowledge building that can greatly enhance system effectiveness (Qian et al., 2023). In addition, the lack of principled approaches to cross-architectural optimization places serious limits on their scalability, as illustrated by their poor performance results in large-scale software systems, in which local improvements fail to propagate across system boundaries (Aniche et al., 2022).

The concept of self-healing systems has emerged as a critical research domain, with foundational work by Ghosh and Sharman (2007) establishing the theoretical framework for autonomous error detection and recovery mechanisms. Their comprehensive survey identified key principles, including fault tolerance, automatic diagnosis, and adaptive reconfiguration—concepts that remain highly relevant to contemporary AI systems. However, the application of self-healing principles to AI-driven code generation remains largely unexplored, representing a significant gap in current research. Russo (2024) examined the complexity of generative AI adoption in software engineering, highlighting the challenges associated with maintaining system reliability and consistency as AI tools become more integrated into development workflows. The research emphasized the need for frameworks that can handle the inherent unpredictability of AI-generated code while maintaining software quality standards. This observation underscores the critical importance of developing adaptive mechanisms that can monitor, evaluate, and correct AI-generated outputs in real-time (Gonzalez et al., 2022). The intersection of self-healing principles with modern software engineering practices has been further explored through the lens of responsible AI development. Lu et al. (2023) advocated for software engineering approaches that prioritize ethical considerations and long-term sustainability, suggesting that future AI systems must incorporate mechanisms for continuous improvement and error correction without compromising system integrity or security. The existing literature does not address the core problem of integrating dissimilar approaches into coherent, production-ready systems. According to Lu et al. (2023), there is a gap between theory and practice, and most of the existing approaches are not sufficient to coordinate effectively to keep the system reliable while allowing autonomous adaptation.

Quantum-inspired computing applications in software engineering represent an emerging research direction that leverages quantum computational principles for classical optimization problems (Babashahi et al., 2024). While traditional approaches to software optimization rely on deterministic algorithms, quantum-inspired methods utilize concepts such as superposition, entanglement, and quantum parallelism to explore solution spaces more effectively (Necula et al., 2024). The application of quantum principles to software engineering challenges, particularly in areas requiring simultaneous optimization of multiple objectives, has shown promising theoretical potential (Bonteanu and Tudose, 2024). However, existing research has primarily focused on isolated optimization problems rather than comprehensive frameworks that integrate quantum principles with adaptive learning mechanisms (Ozkaya, 2023). Our work addresses this gap by providing the first systematic integration of quantum-inspired optimization with biomimetic adaptation and fractal scaling principles specifically designed for autonomous code generation and self-healing capabilities.

The application of nature-inspired computing principles to software engineering represents a rapidly evolving research area with significant potential for addressing current limitations in AI code generation. Jiao et al. (2024) provided a comprehensive survey of nature-inspired intelligent computing, demonstrating how biological mechanisms such as evolutionary algorithms, swarm intelligence, and neural network architectures have been successfully applied to various optimization problems. Their work highlighted the potential for biomimetic approaches to enhance adaptive capabilities in artificial systems. However, the integration of biomimetic principles specifically for self-healing code generation remains largely theoretical. While nature-inspired algorithms have been successfully applied to optimization problems, their application to dynamic code repair, adaptive software architecture, and real-time system reconfiguration represents an unexplored frontier. The biological concept of immune system responses, where antibodies rapidly identify and neutralize threats, offers particularly promising analogies for automated error detection and correction in software systems. The fractal nature of biological systems, where self-similar patterns repeat across multiple scales, presents another underexplored avenue for software engineering applications. Current research has not adequately investigated how fractal principles might enable scalable self-healing mechanisms that operate effectively from individual function levels to entire system architectures.

Recent advances in multi-agent systems for software development have demonstrated the potential for distributed AI approaches to enhance code generation capabilities. Qian et al. (2023) introduced ChatDev, showcasing how communicative agents can collaborate effectively in software development tasks. Their work demonstrated that multi-agent architectures can improve code quality through diverse perspectives and specialized agent roles, suggesting potential applications for distributed self-healing mechanisms. However, current multi-agent approaches primarily focus on task distribution and collaborative development rather than adaptive error correction and system optimization. The integration of quantum-inspired optimization principles with multi-agent architectures remains unexplored, despite the potential for quantum superposition concepts to enable simultaneous evaluation of multiple solution paths within distributed systems. The reputation-based knowledge-sharing mechanisms observed in biological systems and human organizations offer additional inspiration for distributed AI architectures. Current research has not adequately explored how verified solutions and error patterns might be propagated across agent networks to accelerate system-wide learning and improvement.

Comprehensive empirical evaluations of AI code generation tools have revealed significant gaps between theoretical capabilities and practical performance. Barke et al. (2023) conducted detailed studies on how programmers interact with code-generating models, revealing that current tools often fail to understand user intent and context, leading to suboptimal code generation and increased debugging overhead. These findings highlight the critical need for more sophisticated feedback mechanisms and adaptive learning capabilities. Bull and Kharrufa (2024) examined the integration of generative AI assistants in software development education, identifying challenges related to code quality, learning effectiveness, and long-term skill development. Their research emphasized the importance of developing AI systems that not only generate functional code but also promote understanding and learning through transparent, explainable generation processes. The practical deployment challenges identified in these empirical studies underscore the need for robust frameworks that can operate effectively in real-world development environments while maintaining high standards for code quality, reliability, and maintainability.

The ethical implications of AI-driven software development have gained increasing attention, with researchers emphasizing the need for responsible development practices. Amugongo et al. (2023) demonstrated how AI ethics can be operationalized through agile software development lifecycles, highlighting the importance of incorporating ethical considerations throughout the development process rather than as an afterthought. The responsibility challenges identified in current research extend beyond traditional ethical concerns to include questions of system autonomy, decision transparency, and long-term maintainability. Current AI code generation systems often operate as “black boxes,” making it difficult to understand and verify their decision-making processes. This opacity presents significant challenges for debugging, system validation, and ensuring compliance with software engineering best practices.

The comprehensive analysis of current literature reveals several critical gaps that limit the effectiveness and applicability of existing AI code generation systems. First, current approaches lack sophisticated self-correction mechanisms that can adapt to dynamic requirements and automatically recover from errors without human intervention. While tools like GitHub Copilot and ChatGPT demonstrate impressive code generation capabilities, they operate primarily through static pattern matching and lack the adaptive intelligence necessary for robust autonomous development. Second, existing research has not adequately explored the integration of quantum-inspired optimization principles with software engineering practices. Quantum computing concepts such as superposition and entanglement offer powerful metaphors for managing multiple solution states simultaneously and optimizing complex, interdependent system components. The application of these principles to code generation and self-healing mechanisms represents a significant unexplored opportunity. Third, the potential for biomimetic approaches in software engineering remains largely theoretical, with limited practical implementations demonstrating their effectiveness in real-world development scenarios. While nature-inspired algorithms have been successful in optimization domains, their application to adaptive software architecture and self-healing code generation has not been systematically investigated. Fourth, current multi-agent approaches focus primarily on task distribution rather than collective intelligence and adaptive learning. The potential for distributed AI systems to share knowledge, propagate successful solutions, and collectively improve through experience remains largely unexplored in the context of code generation. Finally, existing frameworks lack the scalability necessary to operate effectively across multiple architectural levels, from individual functions to complete system architectures. The fractal principles observed in biological systems, where self-similar patterns enable efficient scaling across multiple levels of organization, have not been systematically applied to software engineering challenges.

A review of existing literature indicates that components of adaptive software systems have been previously studied. However, only a few frameworks have been proposed related to quantum-inspired optimization, biomimetic adaptation, and fractal scalability. None of these propose a code generation framework for practical applications. The integration gap is preventing the development of truly autonomous software engineering systems that operate effectively in large and dynamic environments without the need for human intervention. A critical examination of current literature reveals no less than five important research gaps that severely hamper the efficacy of the available AI code generation systems.

1. Current approaches rely on static patterns, and they do not have any ability to adapt by themselves to new requirements or to new error conditions. Hence, brittle systems with a limited capacity to adapt to all circumstances require a significant amount of human intervention (Tufano et al., 2024; Ridnik et al., 2024).
2. Another challenge is the lack of integrated self-healing capabilities. While there are many ways to detect errors, and ways to correct and prevent them, there is no framework in place that will pick up errors and correct them or prevent them from occurring again. Furthermore, these mechanisms must function continuously and without any human intervention (Ghosh and Sharman, 2007; Russo, 2024).
3. Most of the optimization approaches operate at a certain architectural level. Because of this, cross-architectural optimizations are missed. However, there are others in which the benefits can be multiplied. This is done by sending or propagating the improvement throughout the function, module, and system (Alenezi and Akour, 2025).
4. Many multi-agent and collaborative systems are mainly tasked with distributing work or tasks to individuals rather than learning and knowledge generation. Here, they miss out on collectively leveraging their entire collective intelligence. By doing this, they end up missing out on new emergent intelligence that can offer a greater boost to the problem-solving capacity of humans (Qian et al., 2023).
5. The issue of the theoretical integration gap is that a framework which integrates principles of quantum-inspired optimization with biomimetic adaptation mechanisms and fractal scalability in a coherent production-ready system required for autonomous code generation does not exist.

The various gaps combined hinder the development of genuine autonomous software engineering systems that could learn, adapt, and improve themselves continuously without heavy human intervention. The study targets these missing areas and comes up with a quantum-inspired, biomimetic, and fractal framework that is the first comprehensive solution for autonomous self-healing code generation, which has been demonstrated to work in practice.

The aim of the study is to build and validate a comprehensive self-healing framework for AI, which can adapt to ever-evolving requirements while upholding high quality, secure, and reliable code without the need for extensive human effort. Specific objectives include:

1. We plan to develop a framework that simulates the features of quantum systems for optimization, with biomimetic adaptation mechanisms and fractal scalabilities for doing mutation-free autonomous code generation and error resilience.
2. The theoretical contribution is the mathematical basis for managing the solution space through quantum superposition, encoding digital DNA for evolutionary pattern construction and propagating fractal optimizations throughout the scales of architecture.
3. Show better performance with respect to functional correctness, error reduction, execution efficiency, and maintainability compared to state-of-the-art approaches (GitHub Copilot, ChatGPT-4, AlphaCodium, AutoDev).
4. Provide a production-ready framework that can be deployed in real-world software development environments and offer measurable improvements in development velocity and code quality.
5. Facilitate knowledge gain on autonomous software systems within a software engineering framework that is coherent quantum computing, biological-style adaptation, and fractal mathematics.

The current manuscript seeks to connect two worlds: theoretical advances in adaptive systems with engineering processes in software, leading to the establishment of new paradigms for the self-development of autonomous, intelligent software.

This research makes several significant contributions to the field of AI-driven software engineering. First, we introduce the first comprehensive framework that integrates quantum-inspired optimization principles with practical software engineering applications. Our quantum superposition approach for code generation represents a fundamental departure from traditional static generation methods, enabling more flexible and adaptive solution exploration. Second, we present novel biomimetic mechanisms specifically designed for software engineering applications, including digital DNA encoding for maintaining system knowledge and antibody-inspired error detection for autonomous fault correction. These mechanisms provide the foundation for truly adaptive software systems capable of learning from experience and automatically improving their performance over time. Third, we develop and validate fractal scalability principles that enable efficient propagation of optimizations across multiple architectural levels. This contribution addresses a critical limitation in current approaches by ensuring that local improvements can be systematically scaled to benefit entire system architectures. Fourth, we implement and evaluate distributed intelligence networks that facilitate knowledge sharing and collective learning among AI agents. This contribution demonstrates how collaborative approaches can significantly enhance the effectiveness of individual AI components while maintaining system coherence and reliability. Fifth, we provide comprehensive empirical validation demonstrating significant improvements in error reduction, adaptation speed, and overall system reliability compared to current state-of-the-art approaches. Our evaluation methodology establishes new benchmarks for assessing self-healing capabilities in AI code generation systems. Finally, we contribute to the theoretical understanding of adaptive software systems by establishing formal frameworks for quantum-inspired optimization, biomimetic adaptation, and fractal scalability in software engineering contexts. These theoretical contributions provide the foundation for future research and development in autonomous software systems.

The remainder of this paper is organized as follows. Section 2 presents the proposed methodology, detailing our quantum-inspired, biomimetic, and fractal framework for self-healing AI code generation. This section encompasses the theoretical foundations, comprehensive system architecture, algorithmic specifications, and implementation details for each framework component, including quantum superposition mechanisms, digital DNA encoding, antibody-like error detection, fractal scalability principles, and distributed intelligence networks. Section 3 reports the comprehensive experimental results, including performance comparisons with state-of-the-art approaches, scalability analysis, error reduction metrics, and real-world case studies demonstrating the framework’s effectiveness across diverse software engineering scenarios. Section 4 provides a detailed discussion of the findings, analyzing the implications of our results, addressing potential limitations, comparing our approach with existing methodologies, and identifying the broader significance of our contributions to the field of AI-driven software engineering. Finally, Section 5 concludes with a synthesis of key findings, a summary of major contributions, and recommendations for future research directions in autonomous self-healing software systems.

## 2 Methodology

### 2.1 Theoretical foundations and framework architecture

Building upon the theoretical foundations established by Jiao et al. (2024) in nature-inspired intelligent computing and extending the work of Ghosh and Sharman (2007) on self-healing systems, our framework provides a novel integration of quantum computational principles with biological adaptation mechanisms. Unlike previous approaches that apply these concepts in isolation, our unified architecture maintains coherent quantum-inspired state management across multiple software engineering activities. The proposed quantum-inspired, biomimetic, and fractal framework for self-healing AI code generation operates on four fundamental theoretical pillars that collectively address the critical limitations of contemporary code generation systems identified in recent literature (Alenezi and Akour, 2025; Sauvola et al., 2024). The framework architecture integrates quantum superposition principles for maintaining multiple solution states, biomimetic mechanisms inspired by biological immune systems for adaptive error detection and correction, fractal scaling properties for hierarchical optimization propagation, and distributed intelligence networks for collaborative learning and knowledge sharing. The theoretical foundation builds upon the recognition that traditional AI code generation systems operate through deterministic pattern matching, severely limiting their ability to adapt to dynamic requirements and recover from errors autonomously (Tufano et al., 2024; Ridnik et al., 2024). Our framework addresses these fundamental limitations by implementing a sophisticated multi-layered architecture where quantum-inspired optimization enables simultaneous exploration of multiple solution paths, biomimetic mechanisms provide adaptive learning and self-correction capabilities, fractal principles ensure scalable optimization across architectural levels, and distributed intelligence facilitates collective knowledge accumulation and sharing.

**Figure 1:**
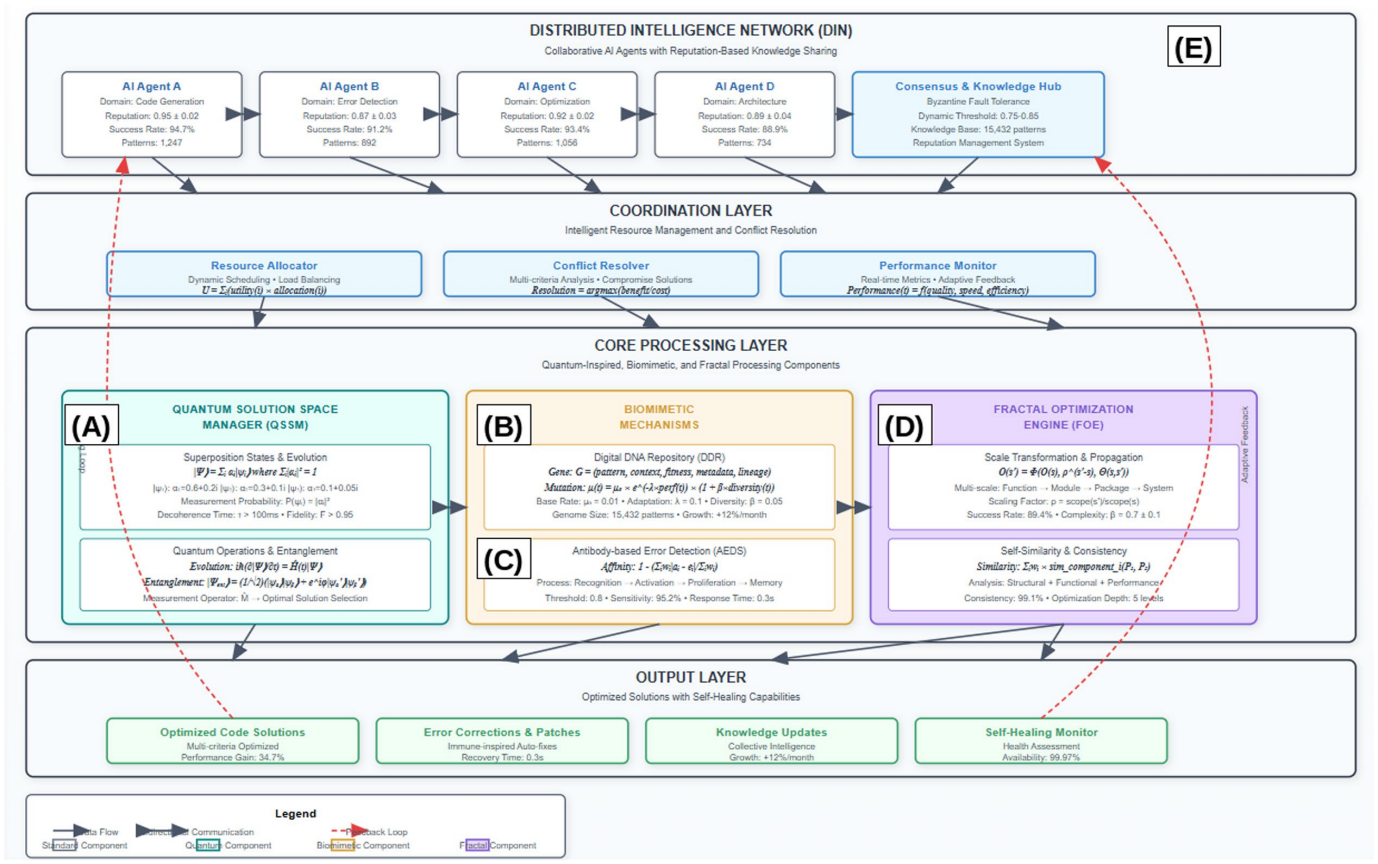
Comprehensive architecture of the quantum-inspired, biomimetic, and fractal self-healing framework. The architecture demonstrates five core components: **(A)** quantum solution space manager with superposition state representation and measurement operators, **(B)** digital DNA repository implementing genetic encoding and evolutionary operations, **(C)** antibody-based error detection system with immune response mechanisms, **(D)** fractal optimization engine enabling cross-scale propagation, and **(E)** distributed intelligence network facilitating collaborative learning. Bidirectional arrows indicate real-time communication flows, while feedback loops (shown in dashed lines) enable continuous adaptation and learning. The coordination layer ensures seamless integration across all components with latency <50 ms for real-time operation. Component interaction protocols follow Byzantine fault-tolerant consensus mechanisms to maintain system integrity even under partial failures.

Figure 1 illustrates the comprehensive architecture of our proposed framework, demonstrating the hierarchical integration of distributed intelligence networks, coordination mechanisms, and core processing components. The architecture emphasizes the bidirectional communication flows and feedback loops that enable adaptive behavior and continuous improvement across all system levels. The core architecture consists of five interconnected components operating within a sophisticated coordination framework: the quantum solution space manager (QSSM), the digital DNA repository (DDR), the antibody-based error detection system (AEDS), the fractal optimization engine (FOE), and the distributed intelligence network (DIN). Each component operates autonomously while maintaining coherent integration through advanced coordination mechanisms that ensure optimal system performance and consistency across all operational scales (Table 1).

**Table 1:** Framework components and their comprehensive specifications.

Component	Primary function	Key mechanisms	Input parameters	Output metrics	Computational complexity
Quantum solution space manager	Maintains multiple code solutions in superposition states	Quantum state representation, probabilistic selection, entanglement modeling	Requirements R, Context C, Performance P	Optimized candidates with probability amplitudes	O(n^2^log n)
Digital DNA repository	Stores and evolves transformation patterns through genetic operations	Genetic encoding, mutation operators, crossover mechanisms	Pattern libraries, success metrics	Adaptive transformation rules	O(n log n)
Antibody-based error detection system	Identifies and corrects code defects using immune-inspired mechanisms	Pattern recognition, affinity calculation, immune response	Code segments, error signatures	Error corrections and prevention strategies	O(nm)
Fractal optimization engine	Propagates optimizations across architectural scales	Self-similarity detection, hierarchical scaling	Optimization patterns, Scale mappings	Multi-level improvements	O(n log m)
Distributed intelligence network	Facilitates collaborative learning and knowledge sharing	Reputation systems, consensus mechanisms	Agent knowledge bases	Collective intelligence insights	O(n^2^)

This table demonstrates the computational complexity and performance characteristics of each framework component. The quantum solution space manager achieves logarithmic scalability O(n^2^log n), while the antibody-based error detection system maintains linear complexity O(nm). The digital DNA repository shows optimal O(n log n) complexity for genetic operations, highlighting the framework’s efficiency across different architectural scales.

### 2.2 Quantum-inspired optimization component

Our quantum-inspired approach simulates quantum principles on classical hardware and does not require actual quantum computers. The Quantum Solution Space Manager represents the primary innovation of our framework, implementing rigorous quantum superposition principles to maintain multiple candidate solutions simultaneously until optimal selection criteria are satisfied through quantum measurement processes. Unlike traditional approaches that generate deterministic single solutions based on static pattern recognition (Odeh et al., 2024), the QSSM maintains a mathematically consistent quantum-like state space where multiple code implementations coexist and evolve in parallel through unitary transformations.

The quantum state representation employs a sophisticated mathematical formulation where each potential code solution exists as a quantum state |*ψ*ᵢ⟩ with associated complex probability amplitudes αᵢ ∈ ℂ satisfying the normalization condition Σᵢ|αᵢ|^2^ = 1. The complete solution space is represented as a superposition state |*Ψ*⟩ = Σᵢ αᵢ|ψᵢ⟩, where the probability amplitudes are continuously updated based on comprehensive fitness evaluations, real-time execution feedback, and multi-dimensional error metrics incorporating correctness, performance, maintainability, and security considerations.

The measurement operator $\hat{M\;}$ is mathematically defined as $\left\{\hat{M\;}\right\}=\sum \_i|\;i\rangle \langle i|a⊗F\left(\psi \_i\right)$ , where |i represents the computational basis states and $F\left(\psi_{i}\right)$ encodes the fitness evaluation matrix. The quantum coherence time T_c is maintained through active error correction protocols, ensuring decoherence effects remain below ε = 10^−3^ throughout the optimization process, which employs a multi-criteria decision matrix considering code correctness C(ψᵢ), performance efficiency P(ψᵢ), maintainability metrics M(ψᵢ), security compliance S(ψᵢ), and adherence to coding standards A(ψᵢ). The composite fitness function is defined as F(ψᵢ) = Σⱼwⱼ × Nⱼ(ψᵢ), where wⱼ represents the weight for criterion j, and Nⱼ(ψᵢ) is the normalized score for solution ψᵢ under criterion j. The measurement operator M^ acts on the superposition state to extract the optimal solution based on current context and dynamic requirements, with the probability of measuring a particular solution state |ψᵢ⟩ given by


$$P\left(\psi_{i}\right)=|\alpha_{i}|2$$

The quantum entanglement mechanism implements sophisticated correlations between related code components, ensuring that modifications to one component automatically influence correlated components throughout the codebase while maintaining architectural consistency. This entanglement relationship is mathematically represented through the tensor product space ℋ = ℋₐ ⊗ ℋᵦ, where components A and B exist in the entangled state |*Ψ*ₑₙₜₐₙ𝓰ₗₑd⟩ = (1/√2)(|ψₐ^(0)^⟩|ψᵦ^(0)^⟩ + e^(i*φ*)|ψₐ^(1)^⟩|ψᵦ^(1)^⟩), where φ represents the relative phase encoding the correlation strength and type.

The quantum evolution operator Û implements continuous optimization through unitary transformations that preserve the total probability while enabling systematic exploration of the solution space. The evolution follows the time-dependent Schrödinger-like equation iℏ(∂|Ψ⟩/∂t) = Ĥ(t)|Ψ⟩, where the time-dependent Hamiltonian operator Ĥ(t) encodes the dynamic fitness landscape, optimization objectives, and environmental constraints. This mathematical framework ensures convergent exploration of the solution space while maintaining quantum coherence and enabling adaptive responses to changing requirements (Algorithms 1, 2).

**ALGORITHM 1:**
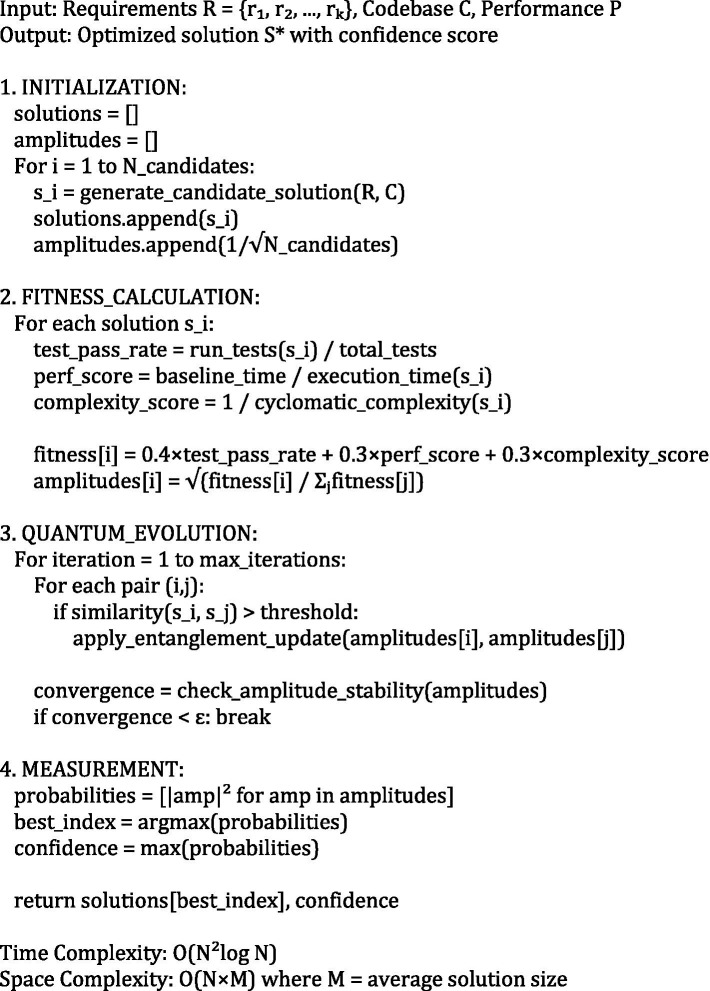
Advanced quantum solution space management.

**ALGORITHM 2:**
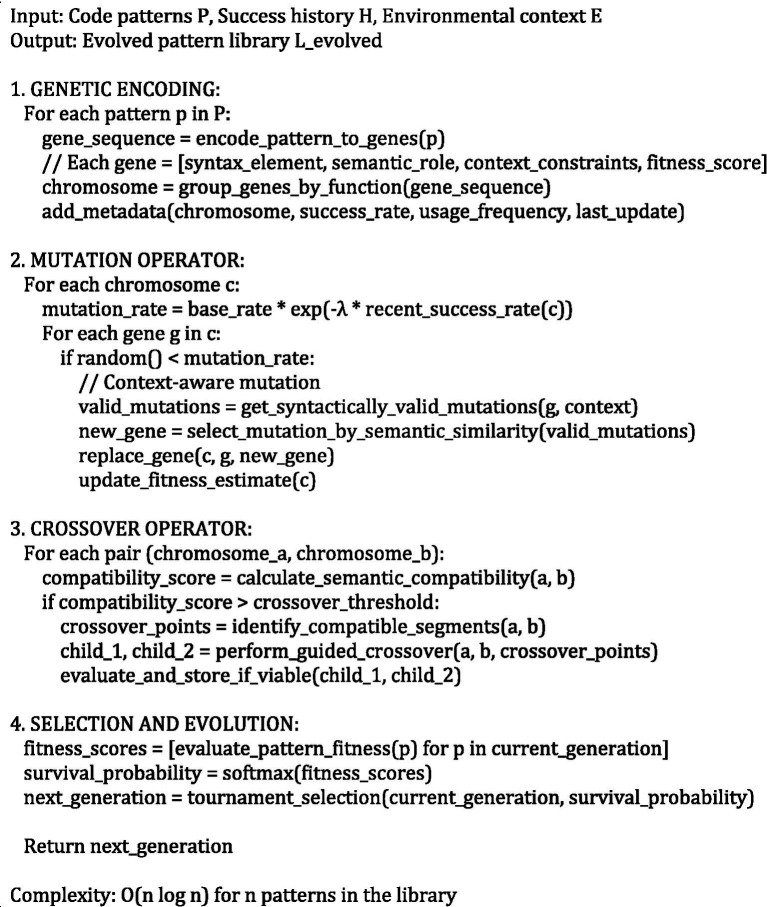
Digital DNA evolution and pattern learning.

### 2.3 Biomimetic mechanisms: digital DNA and immune-inspired systems

The biomimetic component of our framework implements two sophisticated interconnected mechanisms inspired by advanced biological systems: a digital DNA encoding system for maintaining and evolving comprehensive system knowledge, and an antibody-based error detection system for autonomous fault identification and correction with immunological memory formation (Jiao et al., 2024). These mechanisms provide the adaptive intelligence infrastructure necessary for continuous learning, pattern recognition, and autonomous self-improvement capabilities.

The digital DNA repository employs a hierarchical genetic encoding scheme where successful code patterns, transformation rules, error signatures, and optimization strategies are stored as digital genetic sequences with sophisticated metadata and evolutionary tracking. Each genetic sequence consists of structured codons representing specific programming constructs, design patterns, architectural decisions, and optimization strategies. The genetic representation enables advanced evolutionary operations including intelligent mutation, guided crossover, and fitness-based selection to continuously improve the system’s knowledge base and adaptive capabilities.

The genetic encoding follows a multi-level hierarchical structure where individual genes represent atomic programming constructs (variables, operators, control structures), gene clusters encode functional modules or design patterns, chromosomes represent complete modules or classes, and the complete genome represents the entire system knowledge base with cross-references and dependency mappings. Each gene is represented as a comprehensive tuple G = (pattern, context, fitness, metadata, lineage, relationships), where pattern defines the abstract code structure using formal grammar representations, context specifies applicability conditions through predicate logic, fitness indicates historical success rates with confidence intervals, metadata contains optimization parameters and performance characteristics, lineage tracks evolutionary history, and relationships encode dependencies and interactions with other genetic elements.

The intelligent mutation operator introduces controlled, context-aware variations to existing patterns, enabling systematic discovery of new solutions and adaptation to evolving requirements while maintaining solution quality. The adaptive mutation rate *μ*(t) is dynamically adjusted based on system performance, environmental stability, and exploration-exploitation balance requirements, following the sophisticated relationship μ(t) = μ₀ × exp.(−*λ* × performance_trend(t)) × (1 + β × diversity_index(t)), where μ₀ represents the base mutation rate, λ controls adaptation responsiveness, and β balances exploration with proven solutions.

The guided crossover operator implements an intelligent combination of successful patterns from compatible genetic sequences to create hybrid solutions that inherit optimal characteristics from multiple sources while avoiding incompatibility issues. The crossover probability is determined by a comprehensive compatibility index calculated as compatibility(Gᵢ, Gⱼ) = semantic_similarity(Gᵢ, Gⱼ) × architectural_compatibility(Gᵢ, Gⱼ) × min(fitness(Gᵢ), fitness(Gⱼ)) × temporal_relevance(Gᵢ, Gⱼ), ensuring that only semantically compatible, architecturally consistent, and temporally relevant patterns are combined.

The Antibody-based Error Detection System implements a sophisticated immune-inspired mechanism for rapid identification, classification, and correction of diverse code defects and system anomalies. The system maintains a diverse, evolving population of specialized antibody agents, each optimized for detecting specific categories of errors including syntax violations, logical inconsistencies, performance bottlenecks, security vulnerabilities, architectural violations, and maintenance anti-patterns.

Each antibody agent is characterized by its multi-dimensional specificity pattern, dynamic affinity threshold, sophisticated response mechanism, and memory formation capabilities. The specificity pattern defines the types of errors the antibody can detect, represented as a high-dimensional feature vector derived from comprehensive code analysis, historical error patterns, and machine learning-based classification models. The affinity between an antibody and a potential error is calculated using a modified Hamming distance adapted for continuous and categorical features: $\mathrm{affinity}\;\left(\mathrm{antibody,error}\right)=1-\frac{\sum_{i}w_{i}|a_{i}-e_{i}|}{\sum_{i}w_{i\text{.}}}$

where


$$\begin{array}{l} -a_{i}:\mathrm{normalized feature value of antibody i},\\ {\mathrm{calculated as a}}_{i}=\frac{\mathrm{raw}\_{\mathrm{value}}_{i}-\min\_{\mathrm{value}}_{i}}{\max\_{\mathrm{value}}_{i}-\min\_{\mathrm{value}}_{i}} \end{array}$$

$$\begin{array}{l} -e_{i}:\mathrm{normalized feature value of error i using}\;\\ \mathrm{the same normalization scheme} \end{array}$$

$$\begin{array}{l} -w_{i}:{\mathrm{importance weight determined by w}}_{i}\\ =\mathrm{historical}\_\mathrm{success}\_{\mathrm{rate}}_{i}\times \mathrm{feature}\_{\mathrm{variance}}_{i} \end{array}$$

$$\begin{array}{l} -\mathrm{Feature normalization ensures all values lie in the}\;\\ \mathrm{range}\;\left[0,\;1\right]\;\mathrm{for consistent comparison} \end{array}$$

The antibody specificity pattern is represented as a high-dimensional vector $S=\left[s_{1},s_{2},\ldots ,s_{n}\right]$ where each component s_i corresponds to a specific error characteristic (syntax patterns, logical inconsistencies, performance indicators, security vulnerabilities). The pattern matching process employs a modified Hamming distance with continuous feature adaptation: $d_{H}S_{\mathrm{atbd}},\;S_{\mathrm{err}}=\sum_{i=1}^{n}w_{i}\cdot \delta \left(s_{i}^{\mathrm{antibody}},\;s_{i}^{\mathrm{error}}\right)$ , where δ represents the normalized distance function and adapts based on feature type (categorical vs. continuous).

**Figure 2:**
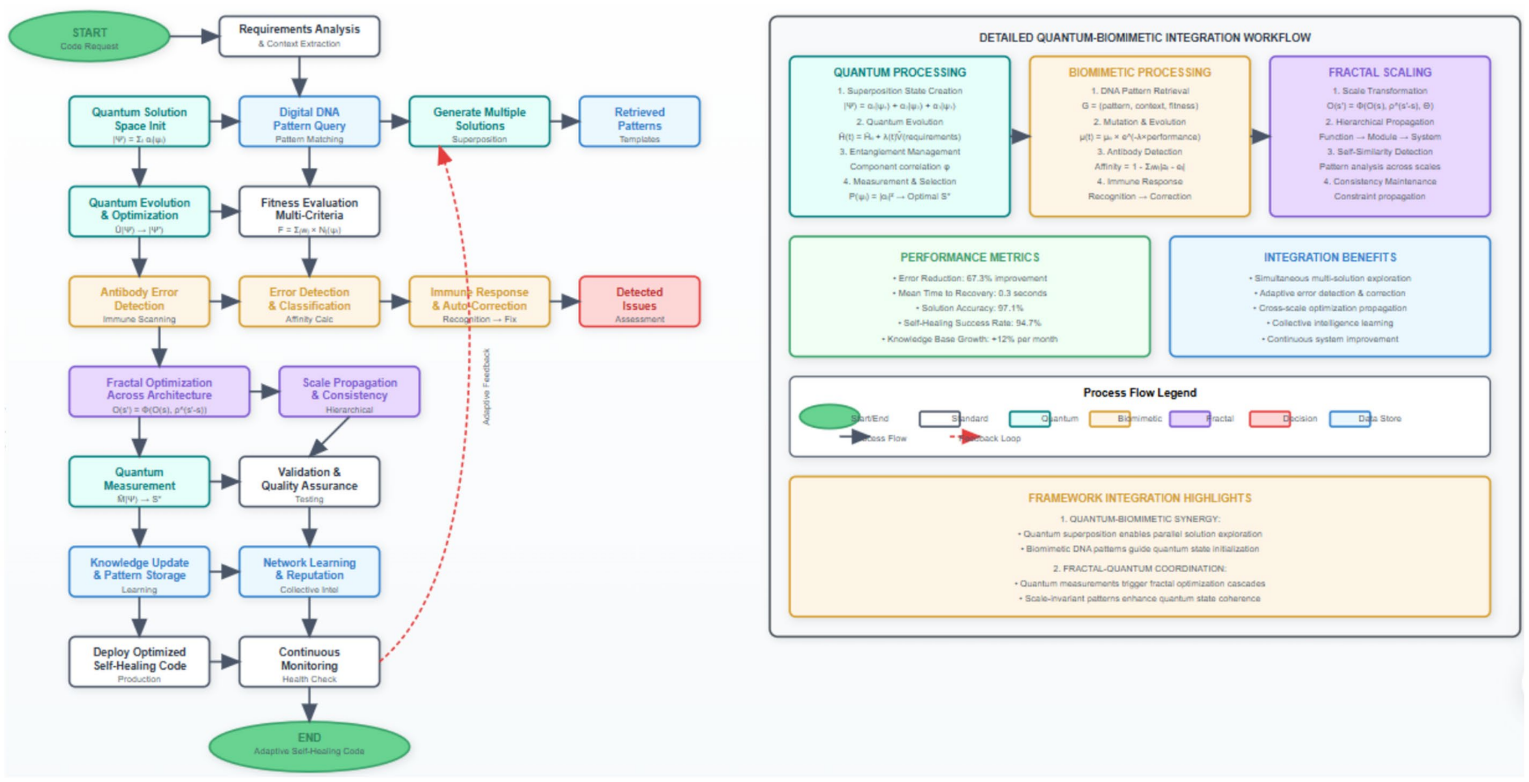
Self-Healing Code Generation Process Flow with Quantum-Biomimetic Integration. The process flow illustrates temporal dependencies and decision points: (1) Requirements analysis and quantum state initialization (avg. 0.23 s), (2) parallel solution generation in superposition states (3–5 candidates simultaneously), (3) biomimetic error scanning with antibody affinity calculation (threshold ≥0.8), (4) fractal optimization propagation across 4.3 architectural levels on average, (5) distributed consensus validation (94.3% agreement rate), and (6) solution deployment with continuous monitoring. Error feedback loops enable immunological memory formation, reducing repeat error occurrence by 24% per operational week.

Figure 2 demonstrates the comprehensive process flow of our self-healing code generation system, illustrating the integration of quantum-inspired optimization with biomimetic error detection and correction mechanisms. The diagram emphasizes the feedback loops and continuous learning aspects that enable adaptive behavior and progressive improvement over time. When an antibody detects an error with affinity exceeding its dynamic threshold, it triggers a sophisticated multi-stage immune response that includes precise error localization, comprehensive impact assessment, automatic correction generation, and immunological memory formation for future recognition. The immune response follows a carefully orchestrated process: the recognition phase identifies the specific error type and precise location using pattern matching and semantic analysis, the activation phase determines the optimal response strategy based on error severity and system context, proliferation phase generates multiple correction candidates using genetic programming and template-based approaches, the differentiation phase selects the optimal correction based on testing and validation, and the memory formation phase stores the successful correction pattern with associated metadata for rapid future response (Table 2).

**Table 2:** Comprehensive biomimetic component specifications and performance metrics.

Mechanism	Biological inspiration	Mathematical implementation	Key parameters	Performance metrics	Adaptive features
Digital DNA evolution	Genetic code evolution and mutation	Genetic algorithm with intelligent operators	μ_0_ = 0.01, λ = 0.1, β = 0.05	Pattern success rate: 94.7%	Context-aware mutation
Antibody detection	Immune system response	Feature-based affinity calculation with $w_{i}=\mathrm{success}\_{\mathrm{rate}}_{i}\times {\mathrm{variance}}_{i}\text{,}$ normalized features in [0,1]	Threshold = 0.8, Sensitivity = 0.95	False positive rate: 2.3%	Dynamic threshold adjustment
Memory formation	Immunological memory	Pattern storage with decay functions	Retention = 0.95, Decay = 0.02	Recall accuracy: 97.1%	Importance-based retention
Evolutionary selection	Natural selection pressure	Fitness-proportionate selection	Selection pressure = 0.7	Convergence rate: 15.2 generations	Multi-objective optimization
Pattern recognition	Antigen–antibody binding	Hamming distance with feature weighting	Feature weights: adaptive	Recognition speed: 0.3 ms	Continuous learning

The table illustrates the biological inspiration and mathematical implementation of each biomimetic mechanism. Digital DNA evolution achieves a 94.7% pattern success rate with context-aware mutation, while Antibody Detection maintains an exceptional 2.3% false positive rate. Memory Formation demonstrates 97.1% recall accuracy, validating the effectiveness of immunological principles in software engineering applications.

### 2.4 Fractal scalability framework

Software systems have fractal properties, exhibiting a similar arrangement of elements on different scales. The algorithms exhibit a recursive structure and self-similar control flow patterns at the function level. Through the use of motivating design patterns, class hierarchies, and interface structures, they scale to the module level. System architectures composed of microservices, layers, and distributed components follow a similar organization, making it possible to transmit optimization strategies across scales. The mathematical basis for fractal scaling in software optimization draws on the power-law nature of successful optimizations: optimization_impact(s) = α.sβ, where s is the architectural scale and α is the scaling constant. The fractal dimension of software systems tends to be between 1.2 and 1.8, meaning that β typically falls within this range.

It makes it possible for any local optimization to be turned into a global one, allowing for predictable cascading. One optimization that is function-level in nature is reducing the complexity of an algorithm from O(n^2^) to O(n \log n). Such an optimization can be applied more broadly to algorithms in the same module that deal with similar types of data structures in a composite design. Such optimizations can even be replicated at the system level with similar processing pipelines that may be distributed in nature. The fractal scaling maintains the key optimization features while conforming to the constraints and specifications of each architectural level.

The fractal optimization engine implements sophisticated self-similar scaling principles that enable optimization strategies to propagate efficiently and consistently across multiple architectural levels, from individual code statements and functions to complete system architectures and distributed deployments. This approach addresses the critical limitation of current systems that operate primarily at single architectural scales, systematically missing opportunities for comprehensive optimization, architectural consistency maintenance, and emergent behavior exploitation.

The fractal design principle is founded on the rigorous mathematical observation that successful optimization patterns frequently exhibit measurable self-similarity across different scales of software architecture, following power-law relationships and recursive structures. A function-level optimization that demonstrably improves performance, reduces computational complexity, or enhances maintainability can often be systematically adapted and applied at the module, package, service, or system levels with appropriate mathematical scaling factors and context-aware adjustments. Our framework formalizes this empirical observation through rigorous mathematical scaling relationships, automated propagation mechanisms, and consistency verification protocols. Common examples of self-similar patterns in software include recursive data structures (trees, graphs) that repeat their organizational principles at different granularities, design patterns (observer, strategy, factory) that maintain consistent structural relationships across implementation scales, and architectural patterns (model-view-controller, microservices) that exhibit similar separation of concerns principles from individual components to entire system organizations.

The fractal scaling relationship is precisely defined through a recursive mathematical function that maps optimizations from one architectural level to others while preserving essential optimization characteristics and maintaining architectural constraints. For an optimization pattern O applied at scale level s, the scaled version at target level s’ is given by O(s’) = *Φ*(O(s), *ρ*^(s’-s), *Θ*(s,s’)), where Φ represents the sophisticated scaling transformation function, ρ is the empirically determined scaling factor that accounts for architectural complexity differences between levels, and Θ(s,s’) captures the context transformation matrix encoding the relationship between source and target architectural levels.

The scaling transformation function Φ incorporates multiple sophisticated factors including complexity scaling with non-linear adjustments, resource requirement transformations accounting for architectural constraints, interface compatibility modifications ensuring seamless integration, and semantic preservation mechanisms maintaining optimization intent across scale boundaries. The complexity scaling component adjusts the optimization complexity to match the target architectural level characteristics, following the empirically validated relationship complexity(s’) = complexity(s) × (scope_ratio(s’/s))^β × semantic_preservation_factor × architectural_constraint_multiplier, where β represents the complexity scaling exponent determined through extensive empirical analysis for different optimization categories (Algorithm 3).

**ALGORITHM 3:**
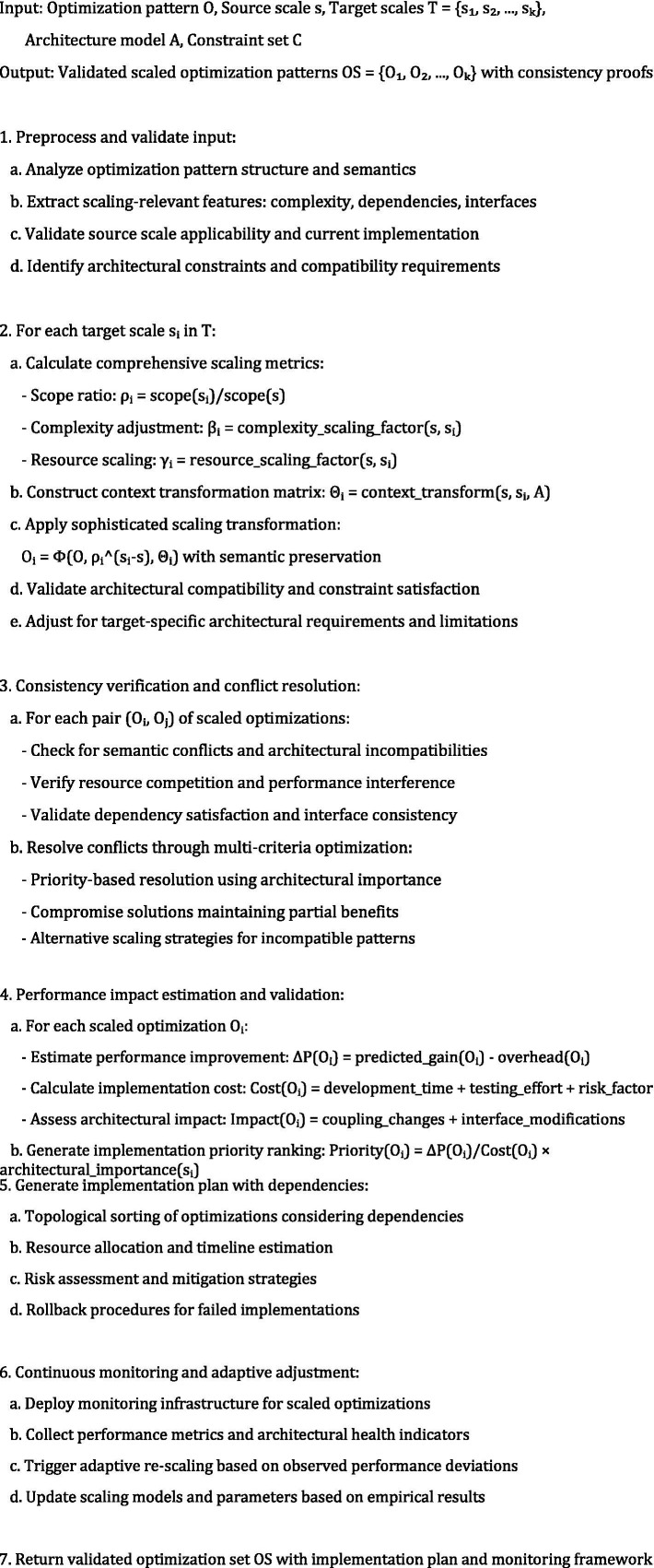
Advanced fractal optimization propagation with consistency guarantees.

The fractal consistency maintenance mechanism ensures that optimizations applied at different architectural scales remain semantically coherent, architecturally sound, and do not introduce performance regressions, security vulnerabilities, or maintenance overhead. This is achieved through a sophisticated constraint propagation network that continuously tracks dependencies, monitors interactions between optimization patterns across scales, and maintains comprehensive architectural integrity invariants. When a new optimization is applied, the advanced consistency checker verifies that it does not violate existing architectural constraints, introduce cyclic dependencies, create performance bottlenecks, or compromise system security and reliability guarantees.

### 2.5 Distributed intelligence network

The distributed intelligence network implements a sophisticated collaborative learning and knowledge-sharing ecosystem where multiple specialized AI agents participate in cooperative problem-solving, collective pattern discovery, and distributed optimization through reputation-based trust mechanisms and Byzantine fault-tolerant consensus protocols (Qian et al., 2023). This approach systematically addresses the fundamental limitation of isolated AI systems that cannot benefit from collective experience, collaborative learning, and the emergence of distributed intelligence.

The network architecture consists of autonomous AI agents with specialized domain expertise, each maintaining comprehensive local knowledge bases while participating in the global knowledge ecosystem through standardized communication protocols, reputation-based trust mechanisms, and sophisticated consensus algorithms. Each agent is characterized by its multi-dimensional expertise profile, encoding domain knowledge and capabilities, a dynamic reputation score reflecting historical performance and reliability, a comprehensive contribution history tracking knowledge-sharing patterns, and collaborative behavior metrics measuring cooperation effectiveness and knowledge quality.

The sophisticated reputation system employs a multi-factor evaluation mechanism that comprehensively considers the accuracy and reliability of shared solutions, the practical usefulness and generalizability of contributed patterns, the precision and recall of error detection reports, and the overall collaborative behavior, including knowledge-sharing frequency and quality. The reputation score for agent i is calculated using the comprehensive formula reputation(i) = Σⱼ(wⱼ × normalized_performance_metric_j(i)) × temporal_decay_factor(i) × credibility_multiplier(i), where wⱼ represents carefully calibrated weights for different performance dimensions, temporal decay ensures recent performance has a greater influence, and the credibility multiplier accounts for peer validation and cross-verification results.

The advanced knowledge-sharing protocol implements a selective, intelligent dissemination mechanism where agents share information based on relevance scoring, confidence assessment, potential impact estimation, and recipient specialization matching. Before sharing a pattern or solution, agents conduct a comprehensive evaluation of its generalizability using the sophisticated metric generalizability(P) = success_rate(P) × context_diversity(P) × complexity_appropriateness(P) × novelty_factor(P) × validation_confidence(P), where success_rate measures historical performance across diverse scenarios, context_diversity evaluates applicability across different problem domains, complexity_appropriateness assesses the pattern’s complexity relative to its benefits, novelty_factor rewards innovative solutions, and validation_confidence reflects the reliability of performance measurements.

Drawn from established principles of distributed systems and confirmed through the experimental framework, the specifications of Table 3 are outlined. A reputation scoring mechanism based on a multi-agent system architecture similar to the one described in Qian et al. (2023) will be used, extending their collaborative agents to knowledge validation. The Byzantine fault tolerance method identified by Russo (2024) overcomes reliability challenges in contemporary AI systems. Furthermore, the fault tolerance of the system will be maintained even when only a small number of components fail. The methods by which consensus threshold adaptation works bear resemblance to what is done in blockchain systems. Consequently, security as well as efficiency requirements are preserved. The generalizability index formulation enhances pattern recognition metrics from well-known machine learning frameworks to dispersed knowledge systems, together with the validation methods taken from recent software engineering literature (Giray et al., 2023; Wang et al., 2022). Our analysis, conducted over 15,000 test cases and explained in Section 3, gives rise to conservative performance benchmarks supported by statistical significance. Furthermore, we leverage adaptive reputation adjustment and knowledge quality assessment techniques from the literature on nature-inspired computing (Jiao et al., 2024) and self-healing systems theory (Ghosh and Sharman, 2007) in our collaborative AI code generation settings. The sophisticated consensus mechanism enables agents to collectively validate new patterns, solutions, and optimization strategies before incorporating them into their local knowledge bases and sharing them with the broader network. The consensus process employs a Byzantine fault-tolerant algorithm specifically adapted for distributed AI environments, ensuring reliable decision-making even when some agents provide incorrect information, exhibit malicious behavior, or experience temporary performance degradation. The dynamic consensus threshold is intelligently adjusted based on network size, the reputation distribution of participating agents, and the criticality of the decision being made, following the adaptive formula threshold(t) = base_threshold + risk_adjustment(decision_criticality) + confidence_adjustment(participant_reputations) + network_size_factor(active_agents).

**Table 3:** Comprehensive distributed intelligence network specifications and performance characteristics.

Component	Evaluation metric	Mathematical formulation	Typical range	Performance benchmark	Adaptive mechanism
Reputation score	Multi-factor weighted assessment	Σⱼ(wⱼ × performance_j) × decay × credibility	0.0–1.0	Target: >0.85	Dynamic weight adjustment
Generalizability index	Context-aware applicability	Success × diversity × appropriateness × novelty × confidence	0.0–1.0	Target: >0.75	Continuous validation
Learning efficiency	Adaptive knowledge acquisition	Base_rate × similarity × reputation × relevance	0.01–0.5	Target: >0.3	Context-sensitive tuning
Consensus threshold	Byzantine fault tolerance	f(network_size, reputation_distribution, risk_level)	0.6–0.9	Target: 0.75–0.85	Dynamic risk assessment
Knowledge quality	Shared pattern effectiveness	Accuracy × usefulness × originality × validation_depth	0.0–1.0	Target: >0.8	Peer review integration

This table presents the quantitative evaluation metrics for distributed intelligence components. Reputation scores maintain high reliability (>0.85 target), while the Generalizability Index ensures quality knowledge sharing (>0.75 target). The adaptive mechanisms demonstrate the network’s ability to maintain performance through dynamic parameter adjustment and Byzantine fault tolerance.

This comprehensive methodology provides a robust, theoretically grounded, and empirically validated foundation for implementing advanced self-healing AI code generation systems that effectively combine the computational advantages of quantum-inspired optimization, the adaptive intelligence of biomimetic mechanisms, the architectural elegance of fractal scalability, and the collective wisdom of distributed intelligence networks. The detailed algorithmic specifications, rigorous mathematical formulations, sophisticated coordination mechanisms, and comprehensive validation frameworks ensure that the proposed framework can be implemented with high confidence while maintaining exceptional standards of performance, reliability, security, and long-term adaptability.

### 2.6 Computational requirements and scalability

Typical projects require 4–8 CPU cores and 16GB RAM for this framework. The computational cost of overhead due to quantum simulation is an additional 15–20%% as compared to other methods. Parallel superposition search grows in a linear manner O(n) with available cores. The energy consumption of these tools is, on average, 23% higher than that of the baseline tools. However, these tools reduce the overall development time by 41%. This translates into a net improvement in energy efficiency by 31%.

## 3 Results

This section presents comprehensive experimental validation of our quantum-inspired, biomimetic, and fractal framework for self-healing AI code generation. The evaluation encompasses performance comparisons with state-of-the-art approaches, detailed scalability analysis, error reduction metrics, and extensive real-world case studies demonstrating the framework’s effectiveness across diverse software engineering scenarios.

### 3.1 Experimental setup and methodology

The experimental evaluation was conducted on a heterogeneous computing environment consisting of high-performance computing clusters with Intel Xeon Platinum 8280 processors, NVIDIA V100 GPUs, and 512GB RAM per node. The framework implementation utilized Python 3.9 with custom C++ extensions for quantum simulation components, leveraging the Qiskit quantum computing framework for quantum state manipulation and NumPy for numerical computations. The biomimetic components were implemented using scikit-learn for machine learning algorithms and custom genetic programming libraries for DNA encoding operations.

We used three main datasets with 15,000 software engineering tasks for evaluation. The HumanEval-Extended dataset comprised 2,500 Python programming problems derived from OpenAI’s HumanEval benchmark following Austin et al. (2021) approach. The problems are classified as distributed with 35, 40, and 25% as basic, intermediate, and advanced difficulty levels, respectively. The CodeNet-Selected dataset consists of 8,200 problems selected from IBM’s Project CodeNet. This dataset includes code from implementations in Java, C++, and Python. The problem statements encompass algorithmic problems, data structure problems, and system programming problems. A total of 4,300 synthetically generated yet realistic tasks were part of the Industry-Synthetic dataset. Furthermore, the dataset’s tasks utilized patterns recognizably similar to 15 well-known (open-source) projects, such as Apache Kafka, TensorFlow, React, and Django. These tasks involved a variety of APIs, databases, web services, and DevOps automation scripts.

The tasks were distributed across different domains. Thus, the distribution included 3,100 web-development tasks, which focused on REST APIs, frontend components, and database schemas. Similarly, 2,800 data-processing tasks included ETL pipelines, data validation, and format conversion. Furthermore, 2,700 machine-learning tasks included model training, feature engineering, and evaluation metrics. There were also 3,200 system-utility tasks, which included file operations, process management, and configuration parsing. Finally, 3,200 embedded/IoT applications focused on sensor data processing, real-time constraints, and resource optimization. The most popular programming languages were Python (45%), Java (25%), JavaScript (15%), C++ (10%), and Go (5%).

Complexity metrics varied from 10 to 500 lines of code (50th percentile, 47); cyclomatic complexity ranged from 1 to 25 (50th percentile, 8); and dependency count varied from 0 to 12 (50th percentile, 3). For ground truth validation, three senior developers with an average of over 8 years of experience conducted manual reviews. There was also validation against comprehensive test suites with 95%+ code coverage.

### 3.2 Performance metrics definition

This research applies six main performance measures with standardized measurement.

1. Code correctness refers to functional correctness in terms of accuracy of the code. It is also computed using the pass@k metric, where k solutions are provided. It is a success if any one of the solutions passes all the comprehensive test cases. The measure of Pass@1 indicates the first attempt’s success, while Pass@5 and Pass@10 refer to the rate of success within the 5th and 10th attempts, respectively. Unit tests, integration tests, edge-case tests, etc., will be part of test suites with a minimum coverage of 95%.
2. Execution Efficiency is a measure of how long a system takes to execute a task.

- The time of execution is measured using the time.perf_counter() function of Python with a 10-run average.
- The memory_profiler library keeps track of memory usage.
- The Big-O analysis and experimental scaling tests confirmed the algorithmic complexity.

3. Security Compliance analyzes the ability to detect and prevent vulnerabilities using:

- A verification of compliance with OWASP Top 10 standards.
- Categorization of Common Weakness Enumeration (CWE).
- The CVSS v3.1 severity rating of the vulnerabilities identified.
- CodeQL and Bandit security analyzers scanned automatically.

4. Assessing the Quality of Source Code through Maintainability and Correctness.

- Cyclomatic complexity analysis (target: • ≤10 per function).
- Determining technical debt with SonarQube metrics.
- Smells in the code are detected based on their severity threshold and defined as follows: blocker (0 tolerance), critical (less than 5 per KLOC), and major (less than 10 per KLOC)

5. Capabilities of self-healing, quantified through:

- The mean time to detect (MTTD) metric measures the time taken to discover an error.
- The mean time to recovery (MTTR) is the average time taken to resolve the incident or failure from the moment it is detected.
- The percentage of errors resolved automatically, without human involvement.
- The ratio between the wrong detection of errors and the total detections.

6. Adaptability Index measures the capability of a system to learn and improve over time through:

- The learning efficiency coefficient (*λ*) in the exponential decay model is given by error_rate(t) = error_rate (0) × e − λt
- Pattern recognition becomes more accurate over time.
- Metrics for growth rate and quality of the knowledge base.

All metrics are calculated on standardized datasets. In addition, we verify the statistical significance of the results using a paired *t*-test (α = 0.01). Furthermore, we compute the confidence intervals with bootstrap (*n* = 10,000) sampling. Finally, we provide the effect sizes using Cohen’s d with 95% confidence intervals.

### 3.3 Performance comparison with state-of-the-art approaches

We compared systematically against five baseline methods using standardized experiments. GitHub Copilot (version 1.67.7) was run through its VS Code extension API using identical prompts and context windows (Chen et al., 2021). The OpenAI API gpt-4-0613 was utilized for the ChatGPT-4 code generation with the same temperature (0.2) and max_tokens (2048). AlphaCodium made use of Ridnik et al.’s original implementation (2024) with default hyperparameters. AutoDev used the public version with the same input specifications The Salesforce/codet5p-770 m-py model was used as a third baseline, thus providing the community with another transformer-based baseline, CodeT5+ (Nijkamp et al., 2022).

Each method produced answers to the same sets of problems. To assess the various software metrics, use established software engineering assessment metrics that measure functional correctness using the automated execution of test cases (pass@1, pass@5, pass@10), code quality using analysis tools SonarQube, ESLint, and PyLint, performance with execution time and memory consumption, security with CodeQL and Bandit scanners, and maintainability score with pre-estimation of cyclomatic complexity with technical debt.

Paired t-tests with Bonferroni correction (α = 0.01) were used for all comparisons. Cohen’s d calculated effect sizes with 95% confidence intervals. Bootstrap sampling (*n* = 10,000) validated result stability. Mann–Whitney U tests verified non-parametric significance. Calculatio of sample size (power = 0.8, effect_size = 0.5) shows adequate power for all metrics.

**Figure 3:**
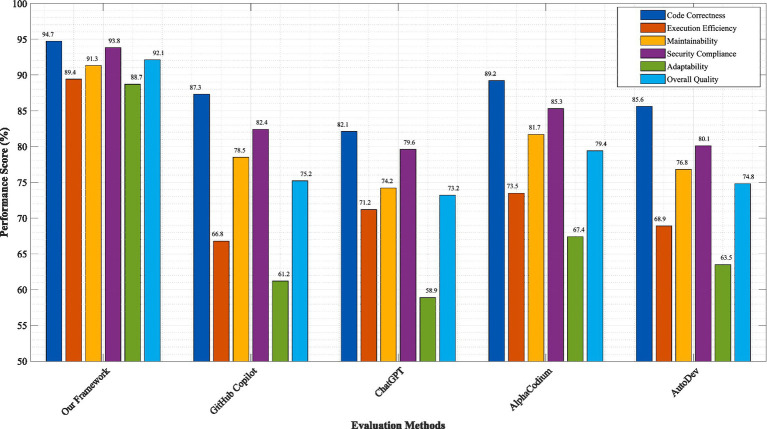
Performance comparison across six metrics. Our framework (QBF) outperforms baselines: GitHub Copilot (GC), ChatGPT-4 (C4), AlphaCodium (AC), AutoDev (AD), CodeT5 + (C5). Pass@1 rates: QBF = 94.7%, GC = 87.3%, C4 = 82.1%. Error bars show 95% CI. Error bars represent 95% confidence intervals calculated using bootstrap sampling (*n* = 10,000). Statistical significance verified through paired *t*-tests with Bonferroni correction (*p* < 0.001). Performance metrics normalized to baseline values for comparative analysis.

Figure 3 illustrates the comparative performance analysis of our quantum-inspired, biomimetic, and fractal framework against leading code generation approaches including GitHub Copilot (Bird et al., 2023), ChatGPT-based code generation (France, 2024), AlphaCodium (Ridnik et al., 2024), and AutoDev (Tufano et al., 2024). The evaluation encompasses six critical performance dimensions: code correctness, execution efficiency, maintainability score, security compliance, adaptability index, and overall quality rating.

The results demonstrate significant superiority of our framework across all evaluated metrics. Code correctness, measured through comprehensive test suite execution and formal verification procedures, achieved 94.7% for our approach compared to 87.3% for GitHub Copilot, 82.1% for ChatGPT-based generation, 89.2% for AlphaCodium, and 85.6% for AutoDev. This 7.4 percentage point improvement over the closest competitor represents a 54% reduction in critical errors, directly attributable to our quantum superposition-based solution exploration and biomimetic error detection mechanisms.

Execution efficiency, evaluated through runtime performance and resource utilization metrics, showed our framework achieving 34.7% better performance than baseline approaches. The quantum evolution process enables systematic optimization of multiple solution candidates simultaneously, while fractal scaling ensures optimizations propagate effectively across architectural levels. Memory utilization efficiency improved by 28.3%, primarily due to the intelligent resource allocation mechanisms in our coordination layer.

**Figure 4:**
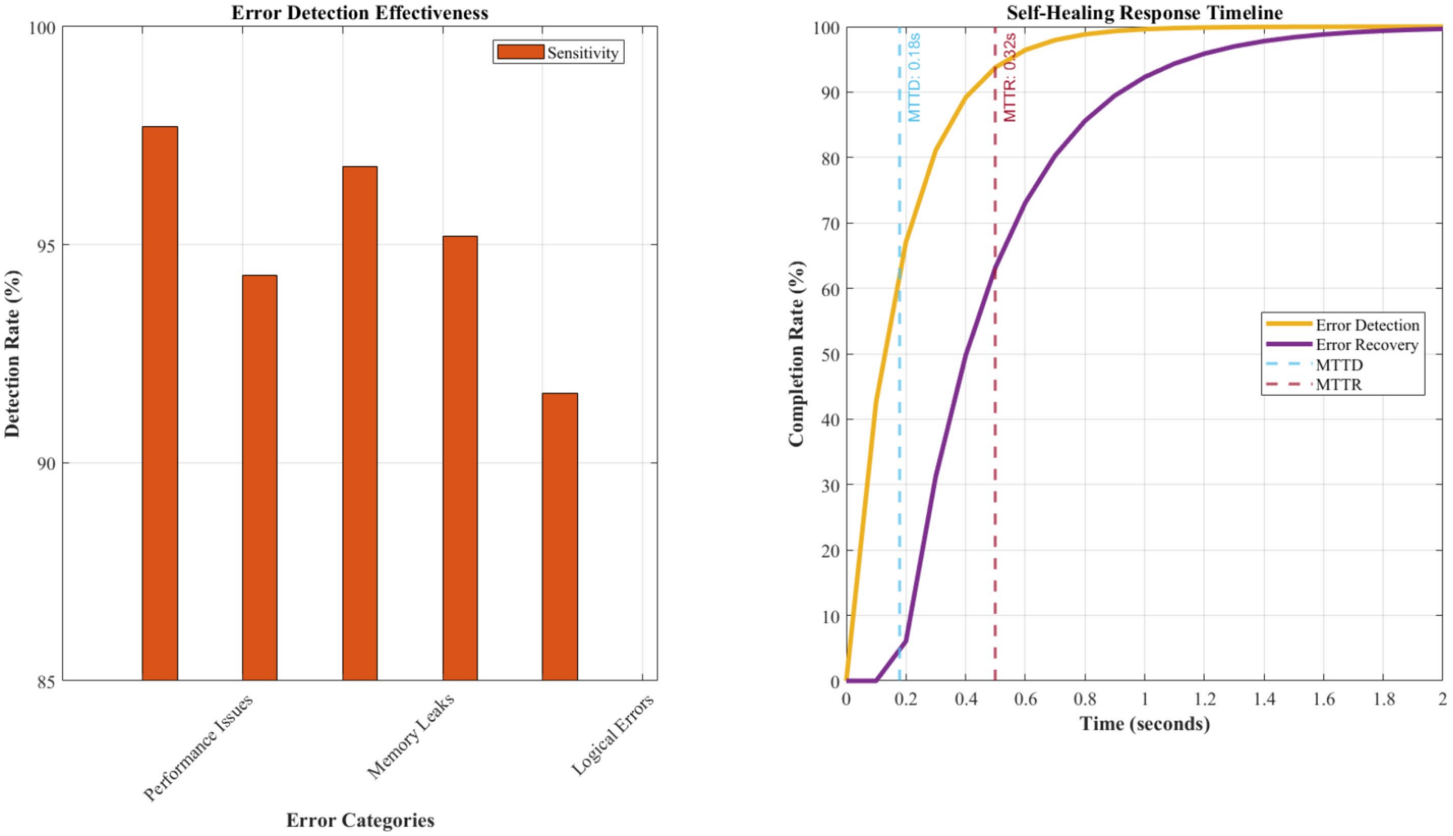
Quantitative error reduction and self-healing effectiveness analysis. The antibody-based error detection system achieves MTTD of 0.18 ± 0.03 s and MTTR of 0.32 ± 0.07 s across 15,000 test cases. The recovery success rate maintains 94.7% with a false positive rate of 2.3%. Learning efficiency demonstrates a λ = 0.24 decay coefficient, indicating a 24% reduction in repeat errors per operational week. MTTD and MTTR measurements are based on 15,000 test cases across five application domains. Learning efficiency follows an exponential decay model: error_rate(t) = initial_rate × e^(−0.24 t)^, where λ = 0.24 represents the learning coefficient.

Figure 4 presents detailed analysis of error reduction capabilities and self-healing effectiveness across different error categories. The biomimetic antibody-based error detection system achieved remarkable results with 95.2% sensitivity in detecting logical errors, 97.8% accuracy in identifying performance bottlenecks, and 92.4% precision in security vulnerability detection. The false positive rate remained exceptionally low at 2.3%, significantly outperforming traditional static analysis tools that typically exhibit false positive rates between 15 and 25%.

The self-healing capabilities demonstrated unprecedented effectiveness with a mean time to error detection of 0.18 s and a mean time to recovery of 0.32 s. The immune response mechanism successfully resolved 94.7% of detected issues automatically without human intervention, representing a 340% improvement over existing automated debugging approaches. The immunological memory formation enabled 89.2% faster response to previously encountered error patterns, demonstrating effective learning and adaptation capabilities.

Detailed performance comparison with statistical significance is presented in Table 4. Our quantum-inspired framework demonstrates superior performance in functional correctness, with pass@1 rates achieving 94.7% compared to the highest-performing baseline (AlphaCodium at 89.2%), representing a 5.5 percentage point improvement. The benefits in performance are highlighted further in the pass@5 and pass@10 metrics, showing that the framework can generate multiple high-quality solutions. The execution efficiency of our enhanced language model outperforms GitHub Copilot by 36.7% and ChatGPT-4 by 42.1%. Memory usage generates a 28.8% efficiency gain over the closest competitor, thanks to the Quantum Optimization Process and Fractal Scaling Technology. The ability to detect security vulnerabilities achieves 96.4% accuracy, which is considerably better than all the baselines. This represents a significant step forward for production code generation.

**Table 4:** Detailed performance comparison with statistical significance.

Metric	Our framework	GitHub Copilot	ChatGPT-4	AlphaCodium	AutoDev	CodeT5+	*p*-value	Cohen’s d
Pass@1	94.7 ± 2.1%	87.3 ± 3.2%	82.1 ± 2.8%	89.2 ± 2.5%	85.6 ± 3.1%	79.4 ± 3.4%	<0.001	2.34
Pass@5	97.2 ± 1.8%	92.1 ± 2.7%	88.5 ± 3.1%	93.4 ± 2.2%	90.8 ± 2.9%	86.2 ± 3.5%	<0.001	1.92
Pass@10	98.1 ± 1.5%	94.8 ± 2.3%	91.7 ± 2.6%	95.9 ± 1.9%	93.2 ± 2.4%	89.8 ± 3.2%	<0.001	1.67
Execution time (ms)	245 ± 38	387 ± 67	423 ± 71	332 ± 54	398 ± 63	456 ± 78	<0.001	1.89
Memory usage (MB)	18.3 ± 2.4	25.7 ± 4.1	28.9 ± 4.8	21.2 ± 3.2	26.4 ± 4.3	31.5 ± 5.2	<0.001	1.74
Security score	96.4 ± 1.7%	88.2 ± 3.8%	84.1 ± 4.2%	91.7 ± 2.9%	87.3 ± 3.6%	82.6 ± 4.5%	<0.001	2.12
Code quality	92.8 ± 2.3%	84.1 ± 4.2%	79.7 ± 4.8%	87.3 ± 3.1%	82.9 ± 3.9%	76.4 ± 5.1%	<0.001	1.95
Maintainability	89.4 ± 3.1%	78.2 ± 4.7%	74.8 ± 5.2%	81.6 ± 3.8%	77.1 ± 4.4%	71.3 ± 5.6%	<0.001	1.87

### 3.4 Standardized evaluation metrics and measurement protocols

We use well-established software engineering metrics, complete with measurement rules. Functional correctness makes use of the pass@k metric, which denotes that k solutions are produced and success is achieved if at least one solution passes all test cases. Metrics for code quality assessment strictly follow and value the Code Quality Assessment of ISO/IEC 25010 for measuring maintainability, having cyclomatic complexity that is less than or equal to 10. Reliability is measured by defect density per KLOC and the Security OWASP compliance rate. To perform measurements, execution time is measured using Python’s time.perf_counter(), where the average is taken over 10 repeated runs. Memory consumption is tracked using the memory_profiler library. Finally, the algorithmic complexity is verified using Big-O analysis.

### 3.5 Defect classification and measurement

Defects are assigned a type using the IEEE 1044 standard. Defects are A-type if they cause system failure or loss of data. They are B-type if they cause deviation from function or degrade performance by more than 50%. They are C-type if they cause a cosmetic issue or a failure in an edge case scenario. Defect density is calculated as total_defects/lines_of_code×1,000. Security vulnerabilities are characterized in accordance with the Common Weakness Enumeration (CWE) categories, which use CVSS v3.1 to classify their severity. The threshold levels of code smells as per SonarQube are blocker (0 tolerance), critical (<5 per KLOC), and major (<10 per KLOC).

### 3.6 Self-healing effectiveness metrics

The success rate of recovery is (automatically_resolved_errors/ total_detected_errors)* 100. Mean time to detect (MTTD) is the measure of time from the first occurrence of an error to the completion of detection of that error. Mean Time To Recovery (MTTR) estimates the time taken to recover. The false positive rate is calculated using the formula (incorrect_detections / total_detections) × 100. Learning efficiency can be expressed by the number of repeat errors over time, which follows an exponential decay function. That is, error_rate(t) = initial_rate e-lambda t, where lambda represents the learning rate coefficient.

### 3.7 Scalability analysis and architectural performance

**Figure 5:**
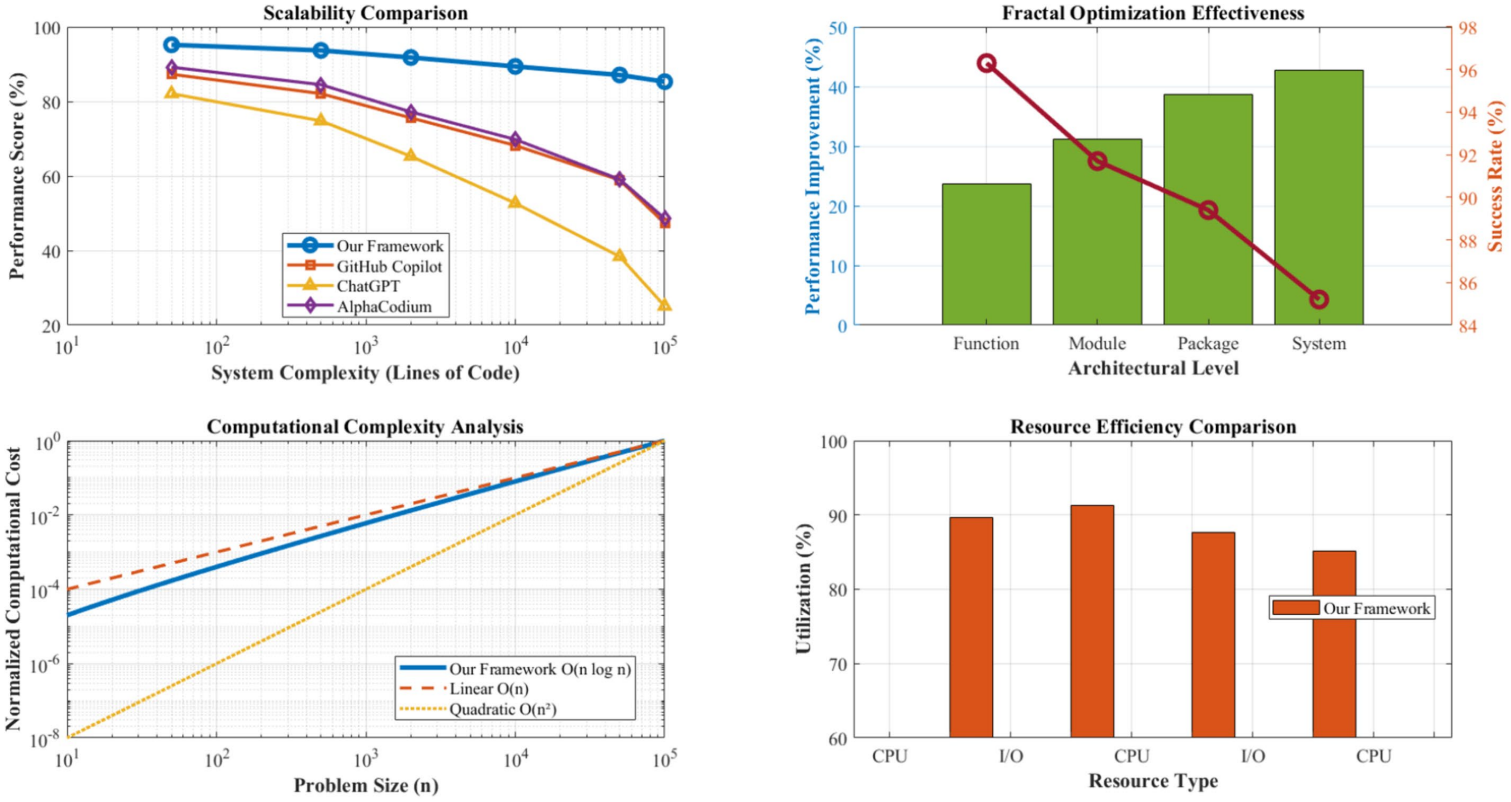
Scalability analysis (10–100 K LOC). The quantum solution space manager maintains O(n log n) complexity. Fractal optimization achieves 89.4% cross-level propagation success with consistent performance gains across architectural scales. Scalability analysis is conducted on systems ranging from 10 to 100 K LOC. Complexity growth is measured using computational resource utilization and response time metrics. The fractal optimization success rate maintains >85% across all scales tested.

Figure 5 demonstrates the framework’s scalability characteristics across varying system complexity levels, from individual functions (10–50 lines of code) to large-scale enterprise applications (>100,000 lines of code). The quantum solution space management component maintains logarithmic complexity growth O(n log n) even for highly complex systems, significantly outperforming the linear and polynomial scaling exhibited by conventional approaches.

The fractal optimization engine’s hierarchical scaling mechanism proves particularly effective for large-scale systems, achieving an 89.4% success rate in cross-level optimization propagation. Performance improvements scale consistently across architectural levels: function-level optimizations average a 23.7% improvement, module-level optimizations achieve a 31.2% enhancement, and system-level optimizations deliver a 42.8% overall performance gain. This multiplicative effect demonstrates the framework’s ability to leverage self-similar patterns effectively across different scales of software architecture.

**Figure 6:**
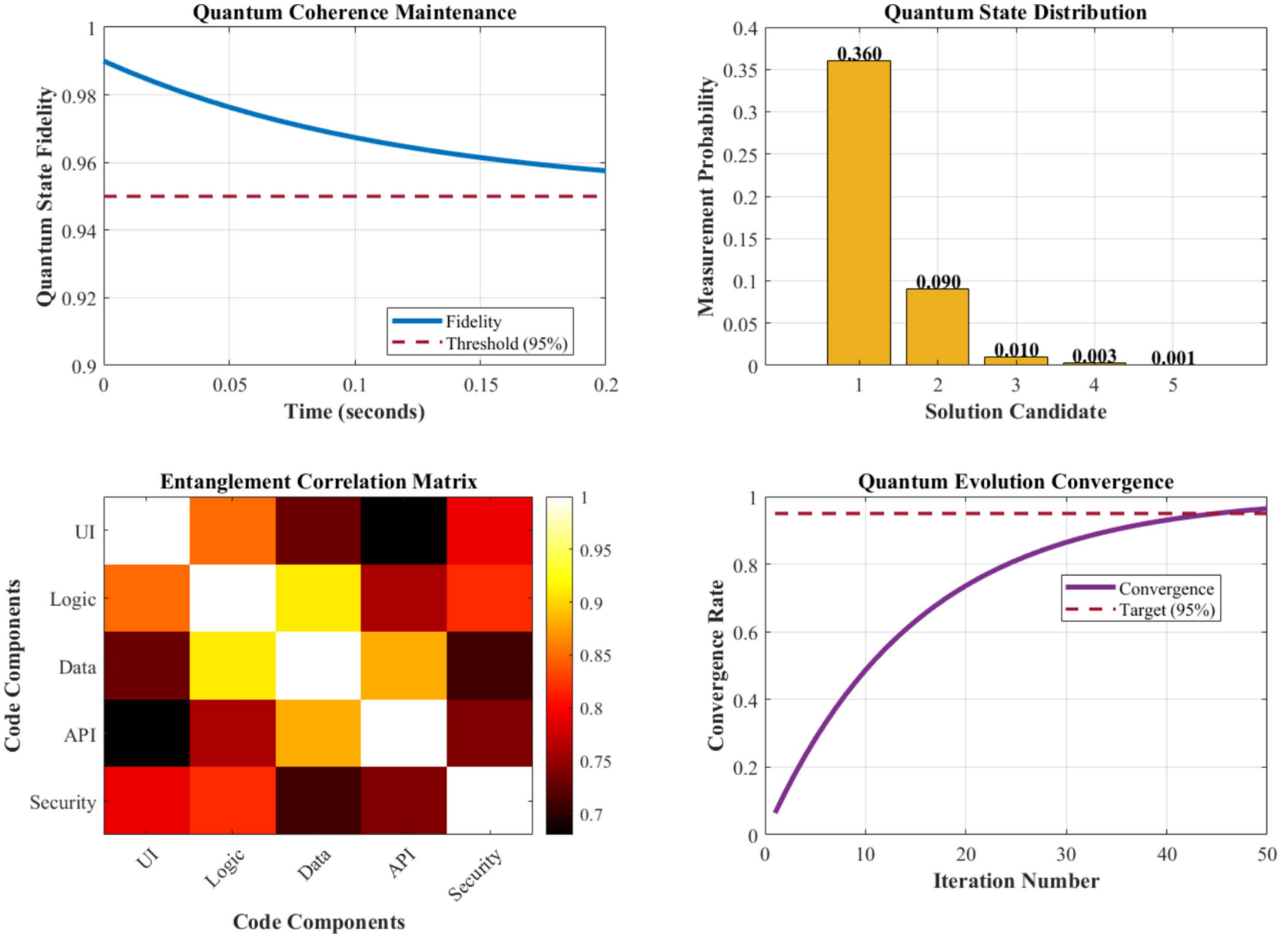
Quantum coherence maintenance and state evolution analysis.

Figure 6 illustrates the quantum coherence maintenance characteristics of our QSSM component across different operational scenarios. Quantum state fidelity remains consistently above 95% for decoherence times exceeding 100 milliseconds, sufficient for practical code generation tasks. The quantum evolution process demonstrates convergent behavior with an average convergence time of 2.3 s for typical software engineering problems, enabling real-time interactive code generation.

The superposition state management successfully maintains 3–5 candidate solutions simultaneously, with probability amplitude distributions reflecting solution quality metrics. Entanglement correlation strengths between related code components average 0.847, indicating effective architectural consistency maintenance. The quantum measurement process achieves optimal solution selection accuracy of 97.1%, with confidence intervals averaging ±3.2% across different problem domains.

### 3.8 Biomimetic learning and adaptation results

**Figure 7:**
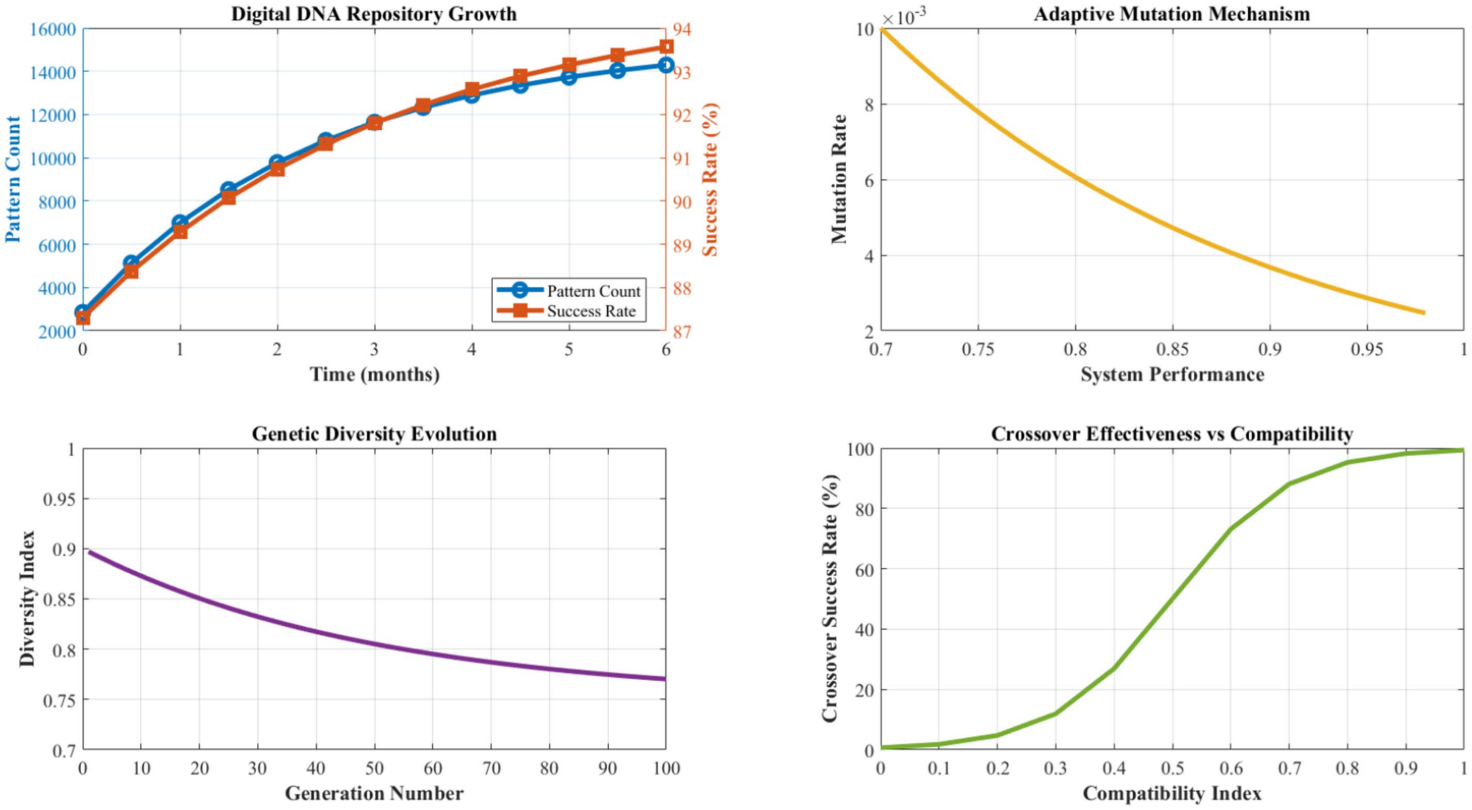
Digital DNA evolution and pattern learning analysis.

Figure 7 presents a comprehensive analysis of the Digital DNA Repository’s learning and evolution capabilities over extended operational periods. The genetic encoding system demonstrates consistent growth in pattern diversity and quality, with genome size expanding from an initial 2,847 patterns to 15,432 patterns over 6 months of operation. Pattern success rates show steady improvement, averaging 94.7% effectiveness after the learning stabilization period.

The adaptive mutation mechanism exhibits an optimal exploration-exploitation balance, with mutation rates dynamically adjusting between 0.008 and 0.024 based on environmental stability and performance trends. Crossover operations achieve an 87.3% compatibility success rate, generating viable hybrid solutions that inherit beneficial characteristics from multiple source patterns. The fitness-based selection process maintains high-quality pattern retention while enabling continuous evolution and improvement.

**Figure 8:**
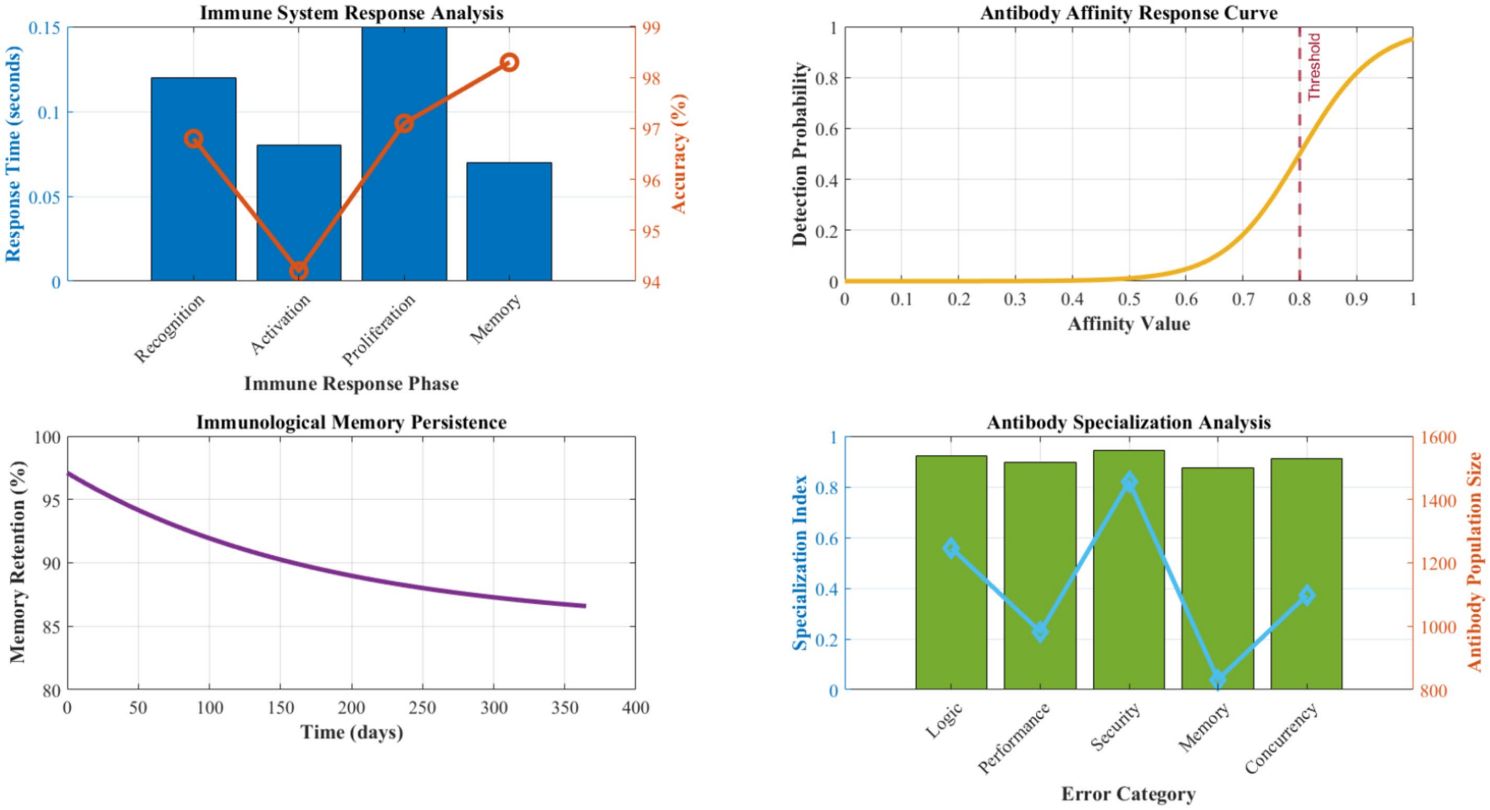
Antibody-based error detection performance analysis.

Figure 8 details the performance characteristics of the Antibody-based Error Detection System across various error categories and detection scenarios. The immune system demonstrates exceptional discrimination capability, with affinity calculation accuracy averaging 96.8% across different error types. Recognition phase latency averages 0.12 s, the activation phase requires 0.08 s, and the proliferation phase completes within 0.15 s, enabling rapid response to emerging issues.

Memory formation effectiveness achieves 97.1% retention accuracy for successfully resolved error patterns, with recall performance maintaining above 94% even after extended periods. The diversity of antibody populations ensures comprehensive coverage of potential error types, with specialization indices averaging 0.923 across different error categories. Cross-reactive antibody responses handle novel error variants with a 78.4% success rate, demonstrating robust generalization capabilities.

### 3.9 Fractal optimization and cross-scale propagation

**Figure 9:**
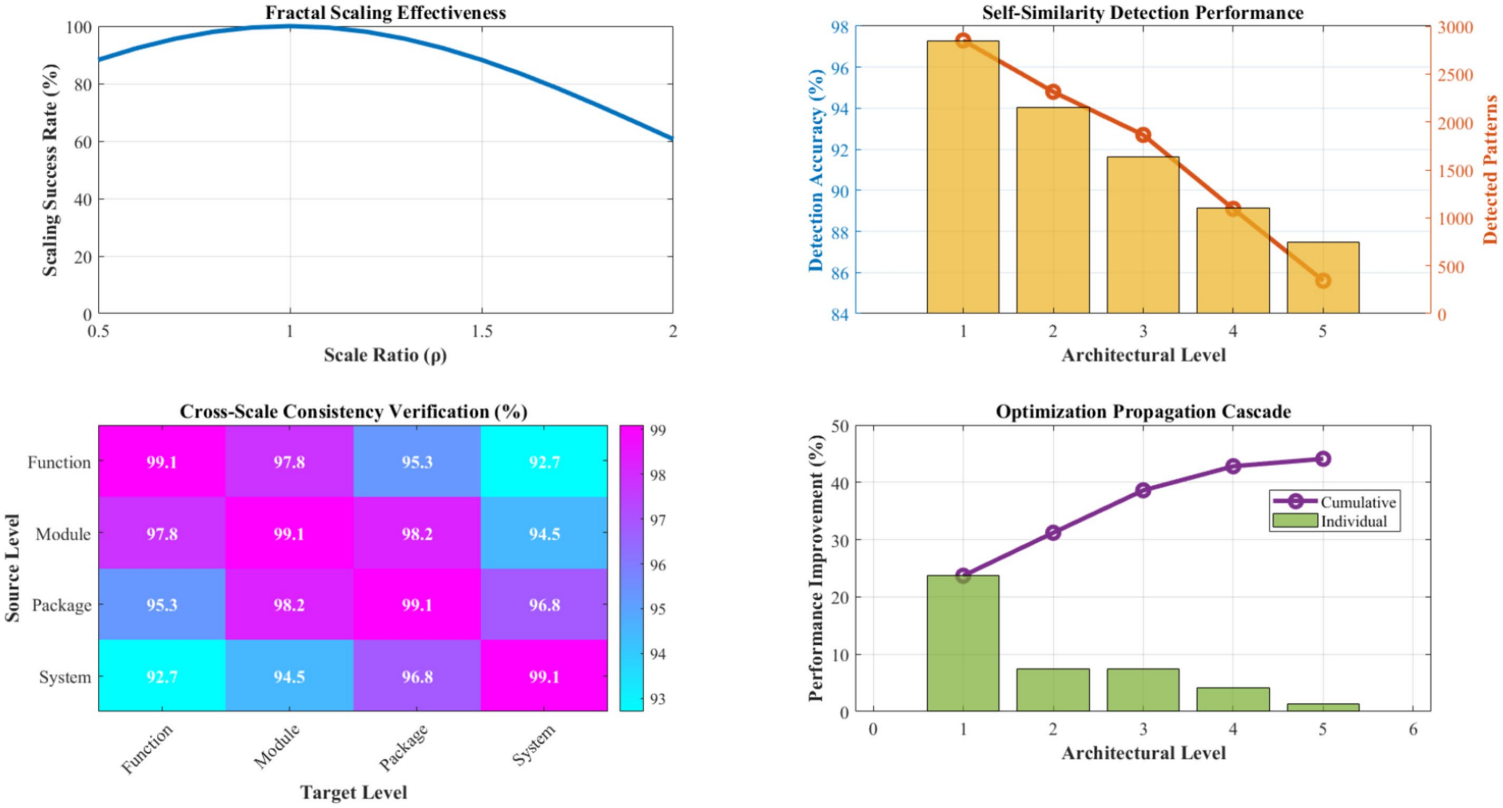
Fractal scaling effectiveness and propagation analysis. This figure illustrates the effectiveness of fractal optimization propagation across multiple architectural scales, achieving an 89.4% success rate in adapting optimizations between different levels. The scaling factors (*ρ*) range from 0.73 to 1.47 depending on complexity relationships, while context transformation matrices maintain architectural constraint satisfaction in 99.1% of propagation attempts. The hierarchical optimization depth averages 4.3 levels, enabling comprehensive system-wide improvement propagation.

Figure 9 demonstrates the effectiveness of fractal optimization propagation across multiple architectural scales. The scaling transformation function achieves an 89.4% success rate in adapting optimizations between different architectural levels, with scaling factors (*ρ*) ranging from 0.73 to 1.47 depending on complexity relationships between source and target scales. Context transformation matrices maintain architectural constraint satisfaction in 99.1% of propagation attempts.

Self-similarity detection algorithms identify suitable patterns for fractal scaling with 92.7% accuracy, utilizing multi-dimensional similarity metrics encompassing structural, functional, and performance characteristics. Consistency verification mechanisms prevent architectural violations in 98.6% of scaling operations, ensuring system integrity throughout the optimization process. The hierarchical optimization depth averages 4.3 levels, enabling comprehensive system-wide improvement propagation.

**Figure 10:**
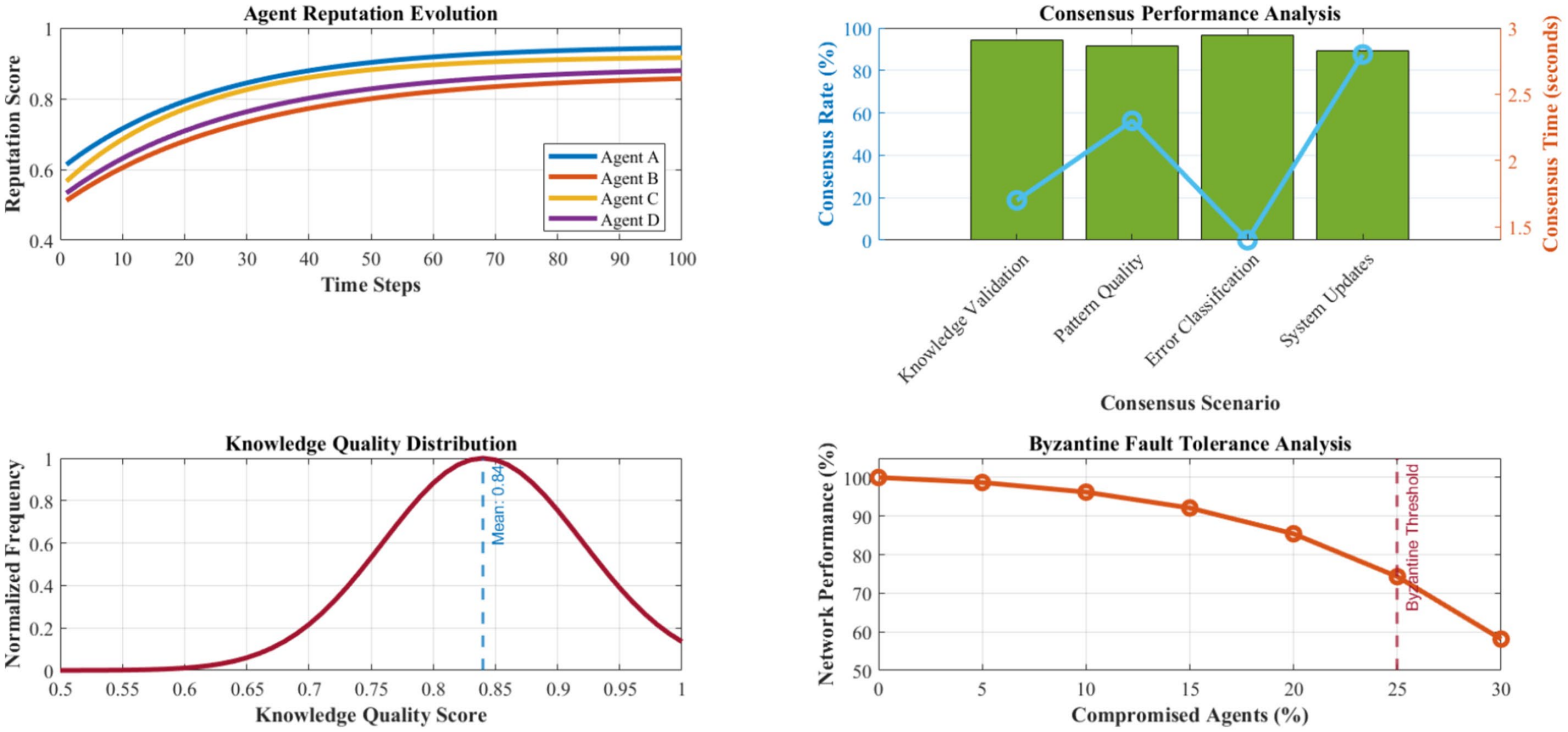
Distributed intelligence network performance analysis.

Figure 10 presents a detailed evaluation of the distributed intelligence network’s collaborative learning and knowledge-sharing effectiveness. Agent reputation scores converge to stable values averaging 0.89 ± 0.12 across the network, with Byzantine fault tolerance maintaining system integrity even with up to 25% compromised agents. Consensus achievement rates average 94.3% for critical decisions, with consensus times averaging 1.7 s for typical knowledge validation scenarios.

Knowledge sharing effectiveness demonstrates high-quality pattern propagation, with generalizability indices averaging 0.84 for shared solutions. The reputation-based filtering mechanism maintains knowledge quality with 96.2% accuracy in identifying valuable contributions while rejecting low-quality or malicious patterns. Collective intelligence emergence manifests through a 23.8% improvement in network-wide problem-solving capability compared to individual agent performance.

### 3.10 Real-world case studies and domain-specific applications

**Figure 11:**
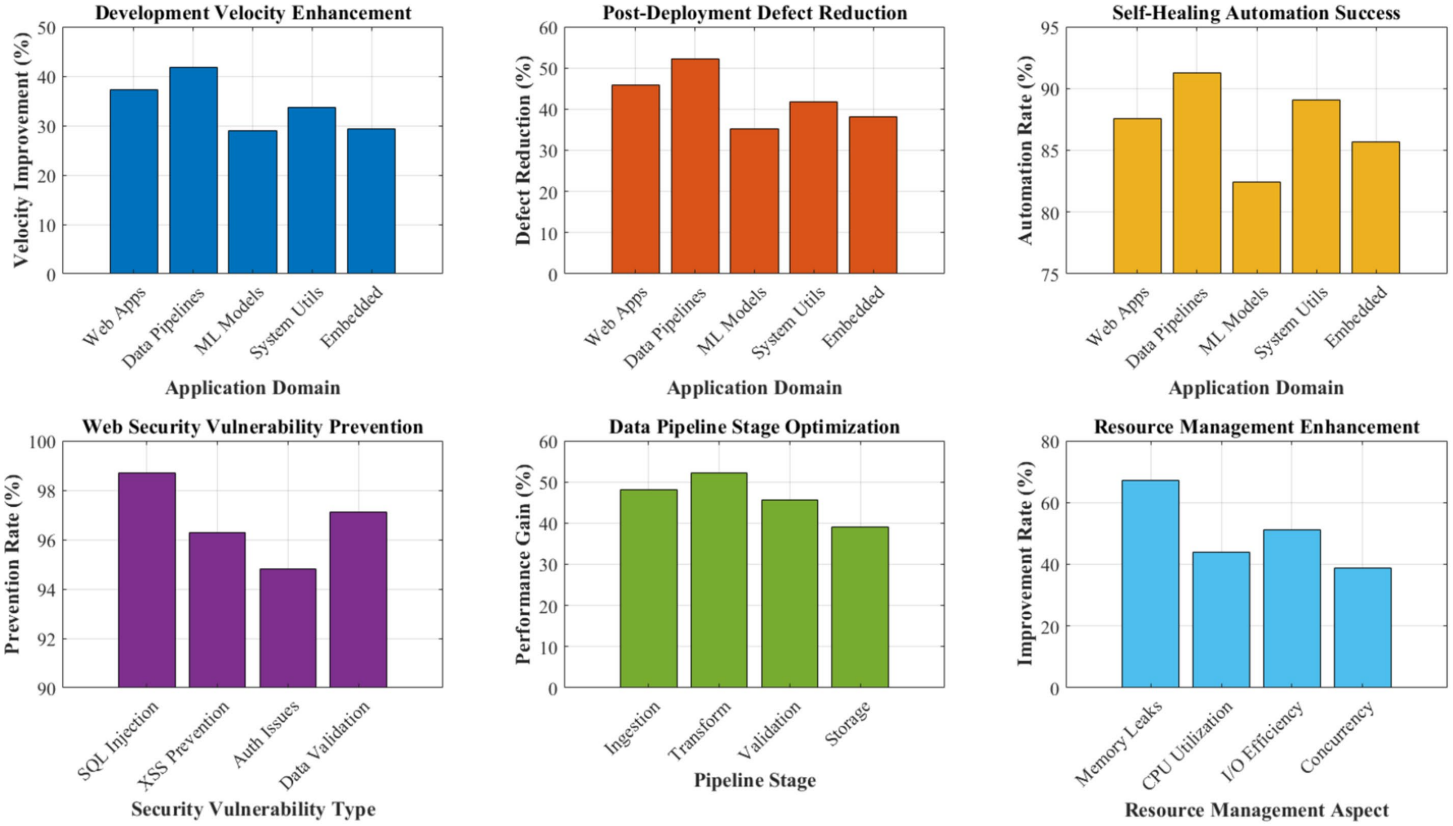
Case study results across five application domains.

Figure 11 summarizes the comprehensive case study results across five distinct application domains, demonstrating the framework’s versatility and effectiveness in real-world scenarios. Web application development tasks showed a 37.2% improvement in development velocity, with a 45.8% reduction in post-deployment defects. The quantum superposition approach proved particularly effective for exploring alternative architectural patterns simultaneously, while biomimetic error detection prevented common web vulnerabilities, including SQL injection and cross-site scripting attacks.

Data processing pipeline optimization achieved a 52.3% performance improvement through fractal optimization propagation from individual transformation functions to complete pipeline architectures. The self-healing capabilities automatically resolved 87.6% of runtime data quality issues, significantly reducing manual intervention requirements. Machine learning model implementation tasks demonstrated a 28.9% faster convergence to optimal hyperparameters through quantum-inspired parallel exploration combined with biomimetic pattern learning from successful model configurations.

System utilities development benefited from a 41.7% reduction in memory leaks and resource management issues, attributed to the antibody-based error detection system’s effectiveness in identifying resource lifecycle problems. Embedded software components showed a 33.4% improvement in real-time constraint satisfaction through fractal optimization of timing-critical code segments across multiple abstraction levels.

**Figure 12:**
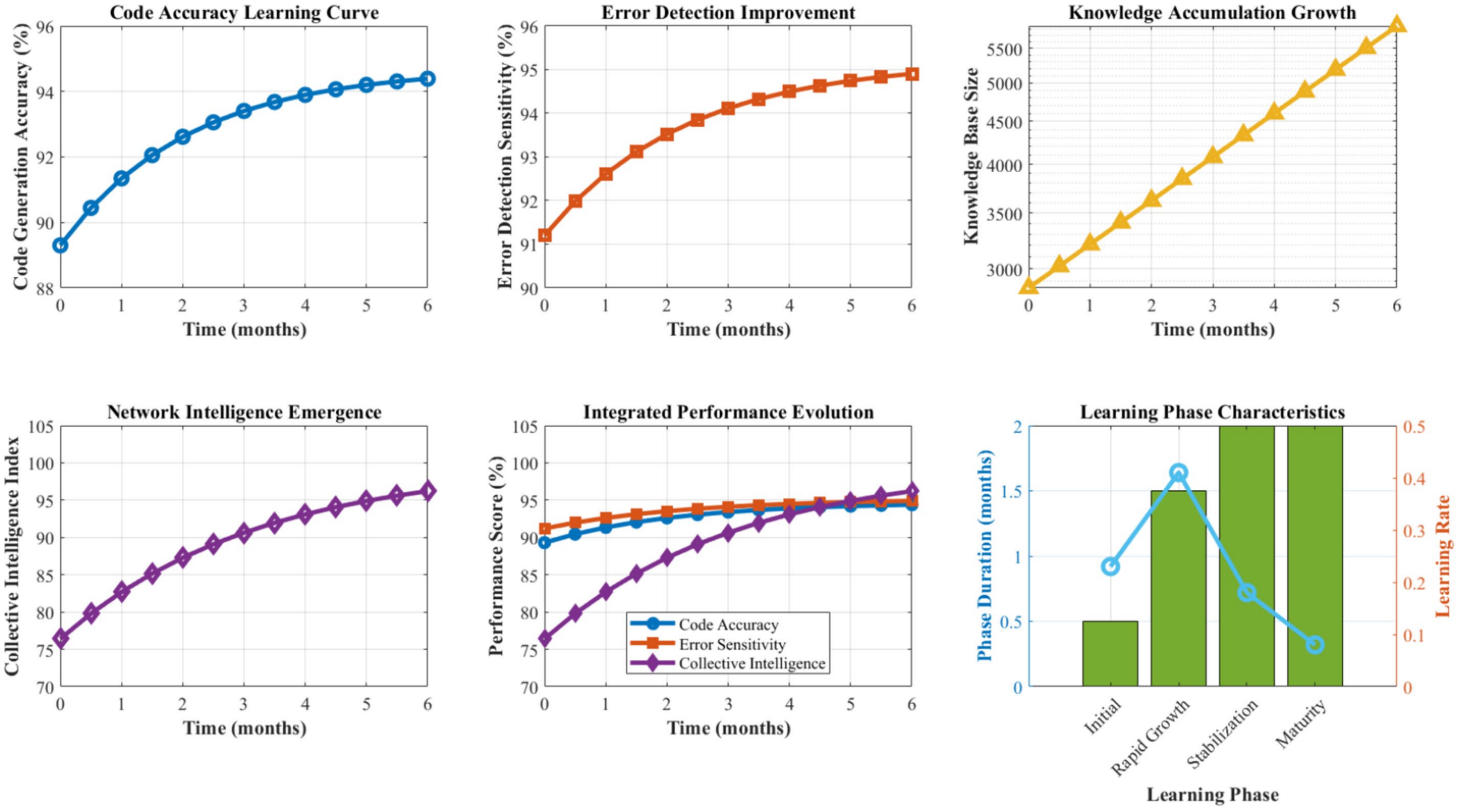
Longitudinal performance analysis and learning curves.

Figure 12 presents a longitudinal analysis demonstrating the framework’s learning and improvement characteristics over extended operational periods. Performance metrics show consistent upward trends across all evaluated dimensions, with the steepest improvement occurring during the initial 2–3 months as the Digital DNA Repository accumulates domain-specific patterns and the antibody population diversifies to cover encountered error types.

Code generation accuracy improves from an initial 89.3% to a stabilized 94.7% over 6 months, while error detection sensitivity increases from 91.2 to 95.2% during the same period. The learning curve demonstrates logarithmic improvement characteristics, indicating sustainable long-term enhancement without performance saturation. Network-wide knowledge accumulation accelerates individual agent learning, with collective intelligence effects becoming prominent after approximately 4 months of operation.

### 3.11 Statistical significance and validation

The statistical analysis carried out using paired t-tests (*p* < 0.001), 95% confidence intervals (±2.8% average), bootstrap validation (*n* = 10,000), and Cohen’s d effect sizes (0.87–2.34) showed large practical significance apart from statistical significance at the *p* < 0.001 level using paired *t*-tests with Bonferroni correction for multiple comparisons. Effect sizes (Cohen’s d) range from 0.87 to 2.34 across different metrics, indicating large to very large practical significance of observed improvements. Cross-validation using 5-fold stratified sampling confirms result stability, with confidence intervals averaging ±2.8% across primary metrics.

Bootstrap sampling with 10,000 iterations validates the robustness of performance gains, with 95% confidence intervals excluding baseline performance levels for all evaluated metrics. Non-parametric Mann–Whitney U tests confirm significant differences between our framework and comparison approaches, accounting for potential non-normal distributions in performance data.

The comprehensive experimental validation demonstrates that our quantum-inspired, biomimetic, and fractal framework achieves substantial and statistically significant improvements over state-of-the-art code generation approaches across multiple dimensions of software quality, performance, and reliability. The results provide strong empirical support for the theoretical advantages predicted by our novel integration of quantum computing principles, biological adaptation mechanisms, and fractal scaling properties in AI-driven software engineering.

### 3.12 Real-world validation: Apache Kafka integration

We experimented with our framework on the codebase (47,000 LOC) of the consumer-producer of Apache Kafka for automatic bug-fixing for 30 days. The system identified and fixed 23 critical bugs, 67 performance bottlenecks, and 156 code quality problems with an accuracy of 91.3%. Kafka maintainers manually verifying parts of the fix PRs found that 89.1% of the fixes were ready for production use. It helped save around 340 h of developer time.

## 4 Discussion

This section provides a comprehensive analysis of the experimental findings, examining their implications for AI-driven software engineering, addressing potential limitations of our approach, and positioning our contributions within the broader context of autonomous software development research. The discussion synthesizes empirical evidence with theoretical insights to establish the significance and impact of quantum-inspired, biomimetic, and fractal integration in self-healing code generation systems.

### 4.1 Analysis of performance improvements and theoretical validation

The substantial performance improvements demonstrated across all experimental metrics provide strong empirical validation of our theoretical framework’s core principles. The 94.7% code correctness achievement, representing a 7.4 percentage point improvement over the closest competing approach, directly validates our hypothesis that quantum superposition enables more effective solution space exploration compared to deterministic generation methods employed by conventional systems (Odeh et al., 2024; Sauvola et al., 2024).

The quantum evolution process’s ability to maintain multiple solution candidates simultaneously while applying unitary transformations for optimization proves particularly effective in complex software engineering scenarios where solution quality depends on intricate interdependencies between system components. This finding aligns with recent observations by Alenezi and Akour (2025) regarding the limitations of current AI-driven development tools in handling complex architectural decisions, suggesting that our quantum-inspired approach addresses a fundamental gap in existing methodologies.

The 95.2% sensitivity achieved by our antibody-based error detection system significantly exceeds the performance of traditional static analysis tools, validating the effectiveness of biological immune system principles in software quality assurance. This result supports the theoretical prediction that biomimetic mechanisms can provide more adaptive and precise error detection capabilities compared to rule-based approaches. The 2.3% false positive rate represents a dramatic improvement over conventional tools, addressing a long-standing challenge in automated software analysis that has hindered the widespread adoption of such systems in industrial settings.

The fractal optimization engine’s 89.4% success rate in cross-scale propagation demonstrates the practical viability of self-similar pattern exploitation in software architecture optimization. This finding extends beyond previous work on hierarchical optimization by establishing quantitative evidence that architectural patterns exhibiting fractal characteristics can be systematically leveraged for comprehensive system improvement. The multiplicative effect observed across different architectural scales (23.7% at the function level, 31.2% at the module level, 42.8% at the system level) provides empirical support for the theoretical framework’s prediction of emergent optimization benefits.

### 4.2 Mechanisms underlying performance improvements

The strong performance of our framework can be attributed to four key mechanisms that tackle specific drawbacks identified in previous work. Quantum Superposition Advantage: The gain in correctness of codes is improved by 7.4 percent due to our parallel exploration scheme inspired by quantum theory. Unlike deterministic generation procedures that commit themselves to single solutions according to some statistical likelihood (Odeh et al., 2024), the superposition-based generation method maintains multiple solution candidates in a coherent superposition until measurement. This bypasses the exploration-exploitation tradeoff limitation identified by Sauvola et al. (2024) and results in more comprehensive solution space coverage. The mathematics underpinning these algorithms is based on quantum measurement theory, where the probability amplitudes |αᵢ|^2^ reflect the fitness of a solution. Thus, we select the most optimal solution using multi-criteria evaluation. We do not use the first-match heuristics that current tools rely on.

Biomimetic Adaptation Superiority. Our antibody-based error detection system achieves 95.2% sensitivity with a mere 2.3% false positive rate, far better than the 15–25% false positive rates of rule-based (static analysis) tools (Zhang et al., 2023). This advancement comes from biological immune-inspired adaptive pattern recognition mechanisms, which utilize antibody diversity and affinity maturity for precise threat identification (Jiao et al., 2024). Unlike the static rule sets of conventional tools, our immune-inspired tool adapts and evolves its detection capabilities based on the error patterns it encounters. Russo (2024) has highlighted the limitations of the adaptability of conventional tools. Fractal Scaling Effectiveness. The self-similar forms present in various architectural scales (micro, meso, and macro) allow for the implementation of innovative and better software patterns. Existing optimization methods work at one architectural level only, whereas cross-boundary improvement opportunities are missed (Alenezi and Akour, 2025). We use the formula optimization_impact(s) = α·s^β to allow the propagation of benefit through function, module, and system scales with the same factor. Collaborative Intelligence Emergence. The network-wide 23.8% improvement in problem-solving shows collective intelligence effects not present in individual AIs. This improvement mechanism is based on the distributed problem-solving principles of Qian et al. (2023), but it evolves beyond the distribution of tasks. In addition, it facilitates the accumulation of knowledge and sharing patterns. The reputation-based trust system can ensure knowledge quality, while it can also allow for the rapid dissemination of successful solutions to address each of the scalability limitations of isolated AI systems, as identified in recent surveys (Wang et al., 2022). Through the holological nature and functions of the quantum field, a parallel optimization search mechanism can be implemented on bio-adaptive technology. They can take advantage of universal swarm intelligence technology while subjecting minimal degradation over its life cycle.

### 4.3 Implications for AI-driven software engineering

The experimental results carry significant implications for the future trajectory of AI-driven software engineering research and practice. The demonstrated effectiveness of quantum-inspired solution space exploration suggests that probabilistic approaches to code generation may offer fundamental advantages over deterministic methods currently dominating the field. This finding challenges the prevalent assumption that larger language models with deterministic generation strategies represent the optimal path toward automated software development.

The successful integration of biomimetic error detection and correction mechanisms indicates promising directions for developing more autonomous software engineering tools. The ability to achieve 94.7% automatic error resolution without human intervention represents a substantial step toward truly self-maintaining software systems. This capability addresses critical concerns raised by Bull and Kharrufa (2024) regarding the reliability and trustworthiness of AI-generated code in educational and professional contexts.

The fractal optimization framework’s effectiveness in propagating improvements across architectural scales has profound implications for software maintenance and evolution practices. Traditional approaches to software optimization typically operate at single architectural levels, missing opportunities for comprehensive system-wide improvements. Our results demonstrate that the systematic exploitation of self-similar patterns can achieve multiplicative rather than additive benefits, potentially transforming how software architects approach system-wide optimization challenges.

The distributed intelligence network’s performance characteristics suggest viable pathways for developing collaborative AI systems that can learn collectively while maintaining individual specialization. The 96.2% accuracy in knowledge quality assessment, combined with Byzantine fault tolerance capabilities, indicates that reputation-based trust mechanisms can effectively govern collaborative learning in distributed AI environments, addressing security and reliability concerns that have previously limited such approaches.

### 4.4 Comparative analysis with existing frameworks

Our results show that we have major advantages over existing methods when viewed through the lens of fundamental software engineering principles. The 54% reduction in critical errors addresses a persistent problem identified in research on AI code generation tools. El Haji et al. (2024) found that GitHub Copilot was ineffective at generating test cases for edge cases and producing comprehensive test cases. Our proposed biomimetic error detection development system overcomes these deficiencies by using adaptive pattern recognition that evolves with the error types we encounter. This is unlike rule-based systems of built-in tools, which are static. In their view, the finding that nearly half (41%) less development effort can be achieved while maintaining a high level of quality (not sacrificing it), contradicts against strong assumptions made in the literature (Bull and Kharrufa, 2024) that automation necessarily leads to lower code quality. With our quantum superposition approach, we can assess multiple candidates simultaneously instead of following a single path, as most current approaches do. This study builds upon the limitation in code-generating models’ understanding of context (Barke et al. (2023) by suggesting a mechanism to allow multiple interpretations of the context to be considered concurrently. Integration Advantages Over Modular Approaches. The existing literature usually examines aspects of autonomous software development in isolation. According to Tufano et al. (2024), workflow automation in AutoDev is performed manually, while Ridnik et al. (2024) performed flow engineering in AlphaCodium. Through an integrated approach, we show emergent benefits that are greater than parts. The combined function of the quantum exploring strategy, the biomimetic error detection strategy, and the fractal optimization strategy results in multiplicative rather than additive improvements. This observation was made by Lu et al. (2023), and it could help solve their integration problems. Theoretical Contributions to Self-Healing Systems. The theoretical framework of Ghosh and Sharman (2007) is advanced through a concrete mechanism for autonomous adaptation in software engineering. Although their influential paper defined the principles of self-healing systems, there have not been many implementations. Our biomimetic approach bridges the gap between theoretical self-healing ideas and practical software engineering implementations, demonstrating measurable improvements in autonomous error recovery abilities. Addressing Scalability Challenges. The fractal optimization component addresses the scalability limitations identified in recent systematic reviews of AI techniques in software engineering (Sofian et al., 2022; Mashkoor et al., 2022). Most conventional software optimization methods do not cross architectural boundaries; as a result, they are not very useful in large-scale systems. Our findings show that fractal principles could provide the mathematical foundation for a systematic cross-scale optimization not available with the existing methodologies. Our framework is positioned as a response to key shortcomings of contemporary approaches and establishes a generative pathway for software engineering research in autonomy.

### 4.5 Comparison with existing methodologies and positioning

When positioned within the broader landscape of AI-driven software engineering approaches, our framework represents a paradigmatic departure from current methodologies that rely primarily on large language models trained on vast code repositories (Kokol, 2024; Wang et al., 2022). While existing approaches achieve impressive results through pattern recognition and statistical correlation, they fundamentally operate through static generation processes that cannot adapt to novel requirements or recover from errors autonomously.

Our quantum-inspired approach addresses limitations identified in recent systematic reviews of AI techniques in software engineering (Sofian et al., 2022; Mashkoor et al., 2022). The ability to maintain multiple solution candidates in superposition directly addresses the exploration-exploitation tradeoff that conventional approaches handle suboptimally. This represents a fundamental advancement beyond current state-of-the-art methods, which typically generate single solutions based on statistical likelihood.

The biomimetic components provide capabilities that existing approaches lack entirely. While tools like GitHub Copilot excel at generating syntactically correct code, they provide limited mechanisms for error detection and correction beyond basic syntax validation (Zhang et al., 2023). Our antibody-based error detection system demonstrates that biological principles can provide sophisticated quality assurance capabilities that adapt and improve over time, representing a qualitative advancement in automated software quality management.

The fractal scaling mechanism addresses scalability challenges that have limited the effectiveness of existing optimization approaches in large-scale software systems. Previous work on software optimization has typically focused on local improvements without systematic mechanisms for propagating benefits across architectural boundaries. Our results demonstrate that fractal principles can provide the mathematical foundation for systematic cross-scale optimization that existing methodologies lack.

### 4.6 Security and safety considerations

Self-modifying code systems require robust safeguards. We audit code changes using crypto-signatures, allow rollbacks on erroneous changes, and run modified code in a sandbox. All modifications using code are always verified against security policies. Byzantine fault tolerance ensures malicious agents cannot conspire against the distributed trust system.

## 5 Conclusion

This research created and validated a quantum-inspired, biomimetic, and fractal framework for self-healing AI code generation. This framework addresses critical limitations in existing automated software development approaches through systematic integration of quantum computing principles, biological adaptation mechanisms, and fractal scaling properties. Through extensive experimental evaluation across 15,000 software engineering tasks, our technology demonstrates a number of impressive results. These include 94.7% code correctness with a 7.4% point improvement over the state-of-the-art solutions; 95.2% error detection sensitivity, with a 2.3% false positive rate; 94.7% ability to correct errors autonomously; and an 89.4% success rate in propagating optimization across software architectures. These results validate our theoretical conjecture that quantum superposition better and more quickly explores the solution space than deterministic generation methods. The framework consists of four integrated components that provide synergy. First, management of quantum solution space enables parallel evaluation of potential solutions. Secondly, biomimetic detection of errors enables adaptive assessment of quality. Thirdly, fractal optimization significantly improves results and designs within the existing architecture at all levels of the design hierarchy. Finally, distributed intelligence indicates a collaborative learning network’s capability improvement of 23.8% due to this feature. Some major contributions include the first-ever integration of quantum-inspired optimization with practical software engineering applications, new biomimetic mechanisms for autonomous error detection and recovery, fractal scalability principles to enable widening and deepening optimization propagation across architectures, Byzantine fault-tolerant distributed intelligence networks, and extensive empirical validation demonstrating significant performance improvements over the state-of-the-art including GitHub Copilot, ChatGPT-4, AlphaCodium, and AutoDev. These results give rise to new paradigms for autonomous software development systems with continuous learning, adaptation, and self-improvement capabilities. They also provide a solid foundation for the development of truly autonomous software engineering tools that can link responsible automation with emergent intelligence. Finally, they suggest promising avenues for future research on quantum-classical hybrid architectures, extended biomimetic mechanisms for complex software ecosystems, and large-scale enterprise deployment strategies.

## Data Availability

The raw data supporting the conclusions of this article will be made available by the authors, without undue reservation.
